# *Cis*-regulatory control of transcriptional timing and noise in response to estrogen

**DOI:** 10.1101/2023.03.14.532457

**Published:** 2023-03-15

**Authors:** Matthew Ginley-Hidinger, Hosiana Abewe, Kyle Osborne, Katelyn L. Mortenson, Alexandra Richey, Erin M. Wissink, John Lis, Xiaoyang Zhang, Jason Gertz

**Affiliations:** 1. Huntsman Cancer Institute, University of Utah, Salt Lake City, UT 84112, USA; 2. Department of Biomedical Engineering, University of Utah, Salt Lake City, UT 84112, USA; 3. Department of Oncological Sciences, University of Utah, Salt Lake City, UT 84112, USA; 4. Department of Molecular Biology and Genetics, Cornell University, Ithaca, NY 14853, USA.

## Abstract

Cis-Regulatory Elements (CREs) control transcription levels, temporal dynamics, and cell-cell variation - often referred to as transcriptional noise. However, the combination of regulatory proteins and epigenetic features necessary to control different transcription attributes is not fully understood. Here, single-cell RNA-seq (scRNA-seq) is conducted during a time course of estrogen treatment to identify genomic predictors of expression timing and noise. We find that genes associated with multiple active enhancers exhibit faster temporal responses. Synthetic modulation of enhancer activity verifies that activating enhancers accelerates expression responses, while inhibiting enhancers results in a more gradual response. Noise is controlled by a balance of promoter and enhancer activity. Active promoters are found at genes with low noise levels, whereas active enhancers are associated with high noise. Finally, we observe that co-expression across single cells is an emergent property associated with chromatin looping, timing, and noise levels. Overall, our results indicate a fundamental tradeoff between a gene’s ability to quickly respond to incoming signals and maintain low variation across cells.

## Introduction

Cis-Regulatory elements (CREs) control the precise spatiotemporal expression of genes across the genome. In addition to a gene’s promoter, many enhancers collaborate to control a single gene’s expression in mammalian cells (ENCODE, 2012; Kundaje et al., 2015; Zhang et al., 2020). External chemical signals often induce changes in cell phenotypes by altering transcription, requiring coordinated gene expression programs. Signal transduction can lead to transcription factor (TF) binding changes and epigenetic modifications at CREs (MacKenzie et al., 2013). For cells to appropriately respond to stimuli, CREs must guide the amount of transcript produced (MacKenzie *et al*., 2013), the timing of transcriptional changes (Kolch et al., 2015; Wei et al., 2016), and the amount of transcriptional variation or noise (Kolch *et al*., 2015; Raj and Van Oudenaarden, 2008; Raser and O’Shea, 2005). While there has been extensive research on the role CREs play in transcription levels, less is understood about the properties of CREs that control gene expression timing and noise.

Temporal regulation of gene expression is an essential attribute of transcriptional control for cellular processes such as cell fate transitions (Basma et al., 2009; Chamberlain et al., 2008; Konstantinides et al., 2022) and responses to signals (Behar and Hoffmann, 2010; Krakauer et al., 2002; Uribe et al., 2021). Specific genes, often termed immediate-early genes, are rapidly activated in response to a signal, while other genes change expression more gradually (Sheng and Greenberg, 1990; Uhlitz et al., 2017; Uribe *et al*., 2021). Genes that show coordinated trajectories are often functionally related, driving diverse phenotypes at different timescales (Gandhi et al., 2011; Krakauer *et al*., 2002; Schnoes et al., 2008; Szustakowski et al., 2007). Previous studies have identified several mechanisms that regulate transcriptional timing. One influential factor is the state of a gene’s promoter. For example, pre-loading of RNA polymerase II (RNAPII) at the promoter is indicative of earlier gene expression responses (Tullai et al., 2007). Additional promoter features associated with early responding genes include TATA motifs at the promoter, a greater number of TF binding motifs, and increased chromatin accessibility (Murai et al., 2020; Tullai *et al*., 2007). Enhancers are also crucial for gene expression timing. Inhibition or deletion of specific enhancers can prolong the time needed for a gene to reach maximal expression without altering final expression levels (Juan and Ruddle, 2003; Simeonov et al., 2017). Stretches of potent enhancers, called super-enhancers, regulate some immediate-early genes (Hah et al., 2015). In contrast, enhancers marked by repressive chromatin marks, termed latent enhancers, exhibit slower activation and are associated with late-responding genes (Ostuni et al., 2013). Generally, relatively little is known about which genomic features in a gene’s cis-regulatory repertoire are important for influencing stimulus-dependent temporal gene responses.

In addition to regulating gene expression timing and levels, CREs control the amount of transcriptional noise. Transcriptional noise is a combination of intrinsic stochasticity and extrinsic variability that cause transcript variation across a population of isogenic cells (Elowitz et al., 2002; Fraser et al., 2021b; Kundaje *et al*., 2015). Cells must regulate transcriptional variation, as both high and low variation have functional consequences. High variation can have benefits, as cells may be more adaptable to changing environments (Pedraza et al., 2018; Wollman, 2018) and more likely to undergo cell fate transitions (Desai et al., 2021; Suderman et al., 2017). Noise may additionally confer the ability of a cell population to produce a diverse output to a single incoming signal (Azpeitia et al., 2020). However, noise can be associated with negative consequences, such as worse cancer outcomes (Han et al., 2016), cancer therapy resistance (Qin et al., 2020; Shaffer et al., 2017), and the ability of cancer cells to metastasize (Fidler, 1978; Nguyen et al., 2016). Both promoters and enhancers can regulate intrinsic noise kinetics and sensitivity to extrinsic noise sources (Larsson et al., 2019). For example, nucleosome positioning and histone modifications at the promoter are important noise regulators (Choi and Kim, 2009; Dadiani et al., 2013; Fraser *et al*., 2021b; Nicolas et al., 2018; Wu et al., 2017), with active histone marks at promoters often associated with low noise (Urban and Johnston, 2018). Additionally, a greater number of transcription factors binding at the promoter may be a basis for greater amounts of noise (Parab et al., 2022). The role of enhancers in controlling mammalian expression noise is less clear. Thermodynamic modeling approaches suggest that multiple enhancers should buffer noise (Hnisz et al., 2017), while experimental evidence shows that super-enhancers are generally associated with noisier expression (Fraser *et al*., 2021b; Wibisana et al., 2022). A remaining challenge is understanding the effect of multiple enhancers in combination with a promoter on expression noise.

To investigate the regulatory control of timing and noise in depth, we focused on the transcriptional response to estrogens. Estrogen Receptor α (ER) is a nuclear hormone receptor activated by estrogens, including endogenously produced 17β-estradiol (E2). In the presence of E2, ER becomes an active TF and regulates the expression of hundreds of genes (Bjornstrom and Sjoberg, 2005). ER is a clinically relevant TF, a potent oncogenic driver for endometrial and breast cancer (Rodriguez et al., 2019a; Stanford et al., 1986), and a well-studied model TF. Upon activation, ER both upregulates and downregulates genes at different timescales (Frasor et al., 2003; Liberzon et al., 2015). Following an estrogen induction, ER activates successive sets of functionally unique genes, as seen in genes related to vascularization, signaling, proliferation, and cell cycle (Jagannathan and Robinson-Rechavi, 2011; Schnoes *et al*., 2008). ER has also been shown to regulate transcriptional noise. Live cell imaging of ER target genes *GREB1* (Fritzsch et al., 2018) and *TFF1* (Rodriguez et al., 2019b) show that ER impacts transcriptional noise by modulating transcriptional kinetics. The temporal, heterogeneous complexity of the ER transcriptional program makes it an ideal model system for studying how CREs regulate transcriptional timing and noise in response to an external stimulus.

To better understand the genomic underpinnings of transcriptional levels, timing, and noise, we analyzed the transcriptional response to E2 using a time course of single-cell RNA-seq (scRNA-seq) in two cell types (human breast and endometrial cancer cells). Feature ranking approaches, using genomic data, revealed important determinants that control these transcriptional attributes. A strong enhancer repertoire was associated with earlier changes in gene expression, which was confirmed using functional perturbation by dCas9-based synthetic transcription factors. Promoter features also regulate timing, such as transcriptional repressor SIN3A being found at the promoters of “Late” genes. We uncovered a balance between enhancers and promoters in regulating expression noise, where strong enhancers drive higher noise and strong promoters are associated with low expression variance. The role of enhancers in timing and noise reveals a tradeoff between expression noise and the ability to respond quickly to incoming signals.

## Results

### Machine learning approach accurately predicts genomic determinants of expression levels

To uncover features of gene regulation that control levels, timing, and noise, pooled scRNA-seq was conducted following 0-, 2-, 4-, and 8-hour E2 treatments in two cell lines: Ishikawa (human endometrial adenocarcinoma) and T-47D (human breast carcinoma) (QC metrics shown in Figure S1A and B). We first set out to identify determinants of mean expression levels by focusing on the 0-hour timepoint (no E2 treatment). Integrated data from publicly available sources (ENCODE, 2012; Shu et al., 2016; Zhang et al., 2016) and experiments conducted for this study were quantified at promoter and enhancer regions. Due to variations in enhancer number and strength across genes, an aggregate enhancer score was used to capture the combined action of multiple enhancers (see methods) (Figure S1C). Genomic features were ranked by importance for classifying low (bottom 20% of genes), medium, and high (top 20% of genes) expression levels using the Boruta algorithm for feature selection (Kursa and Rudnicki, 2010b), which has been previously used to uncover determinants of expression noise in *drosophila* (Sigalova et al., 2020). For feature ranking, we grouped genes from both cell types to find mutual predictors, with the expectation that there are common underlying mechanisms for transcriptional control.

**Figure 1.**
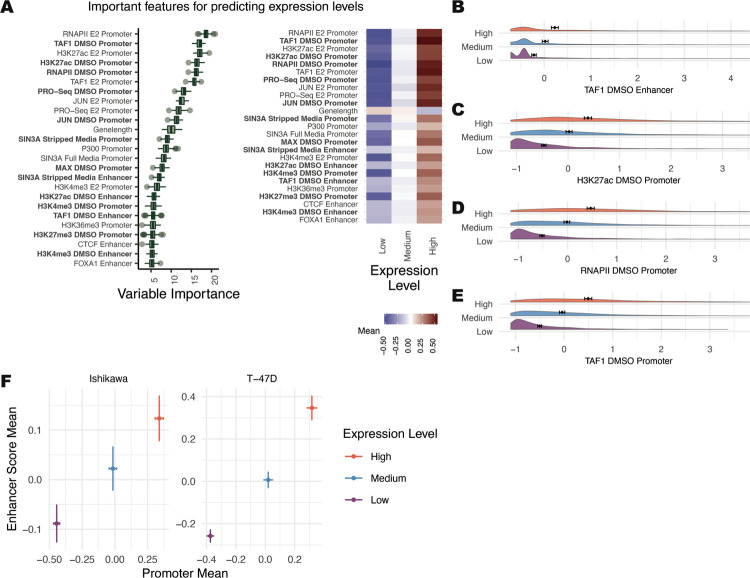
General transcription factors are the strongest predictors of gene expression levels. (A, left) Boruta feature ranking of genomic features shows importance of a feature for predicting mean levels. (A, right) Average signal intensity for each genomic dataset, grouped by mean expression levels is shown. Datasets shown in bold were performed in the absence of ER activation. (B-E) Distributions of the top 4 most important ranked features in the DMSO condition, separated by mean expression levels, show higher signal for “High” expression groups. X-axis represents Z-scores and error bars show the mean ± 95% confidence intervals. (F) Mean enhancer score signal for all Boruta confirmed features vs. mean promoter signal across all confirmed features is shown where error bars show the mean ± 95% confidence intervals.

Elements of the pre-initiation complex and H3K27ac at the promoter ranked as the most important predictors for transcript levels (Figure 1A left). These features had stronger promoter signals for higher expressed genes (Figure 1B–E), in agreement with previous literature (Schier and Taatjes, 2020; Wang et al., 2008). Important factors at promoters and enhancers showed a general trend of increased signal for highly expressed genes (Figure 1A, right). Our dataset is strongly biased toward activating transcription factors and activating histone marks. Plotting the average promoter intensity compared to the average enhancer score across all confirmed datasets verifies that strong promoters and active enhancers are associated with higher gene expression levels (Figure 1F). Overall, the Boruta approach was successful at identifying known predictors of transcript levels.

### Analysis of temporal trajectories indicates that CRE features control estrogen response timing

**Figure 2.**
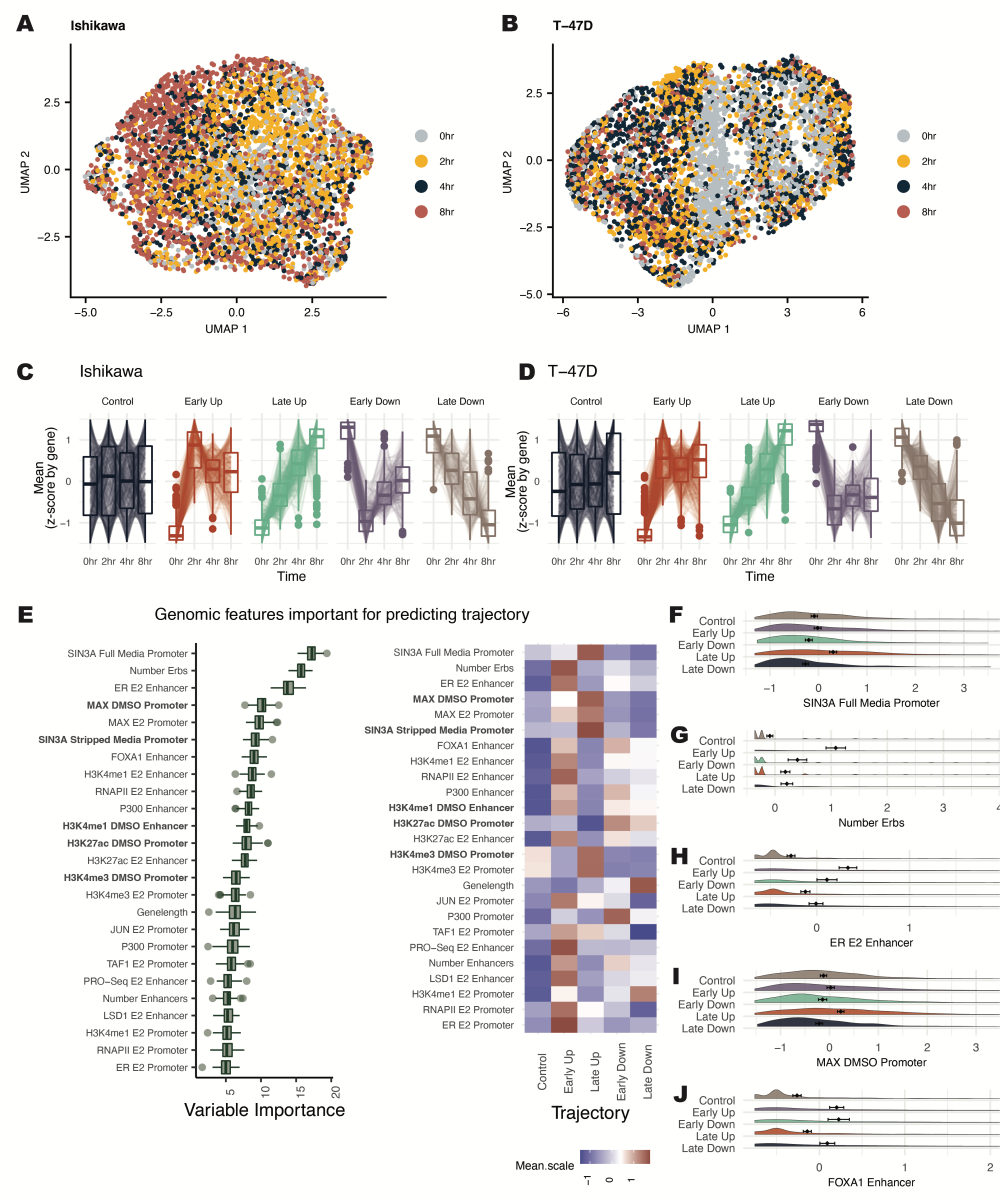
Feature ranking for predicting expression trajectories reveals SIN3A and multiple ERBS as strong predictors. (A-B) UMAP dimensionality reduction plots for (A) Ishikawa and (B) T-47D show temporal progression of cells treated with E2. Each point represents a cell and colors show timepoints post 10nM E2 induction. (C-D) Z-scores for each gene across 4 timepoints are shown for classified trajectories of gene expression in (C) Ishikawa and (D) T-47D cells. (E) (left) Based on Boruta ranking, the top 25 most important features are shown for classifying gene trajectories. (right) Heatmap displays the average signal by trajectory for each predictor. Datasets shown in bold were performed in the absence of ER activation. (F-J) Distribution of signal (Z-score) of the most important features for predicting temporal trajectories is shown.

scRNA-seq gene expression data following 0-, 2-, 4- and 8-hours of estrogen treatment was used to uncover genomic predictors of temporal transcription responses to E2. Using dimensionality-reduction UMAP plots, the temporal progression of the E2 response in single cells can be observed (Figure 2A and B). Based on a Wilcoxon rank-sum test (Bauer, 1972), there were approximately 2000 differentially expressed genes for each cell type between any E2 timepoint compared to the 0-hour control. scRNA-seq summed counts showed high concordance with previously conducted bulk RNA-seq (Figure S2A and B) (Gertz et al., 2013). Compared to bulk RNA-seq, there are more differential genes with higher expression (Figure S2A and B) and lower fold changes (Figure S2C and D), likely due to the increased statistical power of scRNA-seq for calling differential expression of highly expressed genes. scRNA-seq can therefore be a valuable tool to capture subtle changes in gene expression following E2 treatment.

Based on the timepoint at which a gene is differentially regulated, genes were classified into temporal response trajectories for up- and down-regulated genes. One class of genes rapidly changes expression in response to E2 (termed Early genes), while another class changes more gradually and takes longer to reach a maximum response (termed Late genes). A representative set of Control genes was also randomly selected using stratified sampling to mirror mean levels found in differential genes (Figure 2C and D). Early responding genes have a significant initial response to E2 by 2 hours, then return toward baseline for both up- and down-regulation, consistent with previous reports of pulse-like expression in immediate-early genes (Iyer et al., 1999). In contrast, genes classified as Late show a slow and steady response over time (Figure 2C and D). As reported previously (Gertz *et al*., 2013), these gene expression changes were mainly cell-type specific. However, genes with Up trajectories are more conserved between cell types than genes with Down trajectories (Figure S2E) (p-value=0.013; t-test).

The Boruta algorithm was used to identify predictors of temporal trajectories. SIN3A signal at the promoter was most predictive of gene expression trajectory and is associated with Late Up genes (Figure 2E and F). MAX, which is known to repress genes in a complex with SIN3A (Baudino and Cleveland, 2001), was also classified as important and enriched at promoters of Late Up genes (Figure 2I). The number of ER binding sites (ERBS) that loop to the promoter and the ER signal at enhancers were the next most important features (Figure 2E). These two features are most significant for Early Up genes (Figure 2G and H), consistent with previous results showing that ER is an enhancer-binding protein (Droog et al., 2016). A higher number of enhancers is enriched for genes that respond Early (Figure S2G). However, specific proteins (e.g., FOXA1) (Figure 2J) are more balanced between Early Up and Early Down genes than other factors (e.g., ER) that show preferential binding to Early Up genes. Together, these results suggest that the number of enhancers plays a prominent role in the temporal response of genes, but transcription factors at these sites, such as ER, help control the direction of gene expression changes. An example of an optimal decision tree was computed to examine a potential hierarchy of factors determining a gene’s temporal response (Figure S2F). This decision tree shows how SIN3A is the primary separator of genes into Late Up gene trajectories and may take precedence over the ER signal. Overall, Boruta analysis of temporal trajectories uncovers unique factors that may regulate transcription response timing and shows the association of multiple ER-bound enhancers with a rapid up-regulation in response to E2.

### Functional perturbation of CREs alters temporal responses

To test the functional relationship between CREs and transcriptional response timing, dCas9-based activators and repressors were used to modulate the genomic activity of regulatory regions. Gene expression responses were then measured during 8-hours of E2 treatment in Ishikawa cells. A SID(4x)-dCas9-KRAB construct was used for repression (Carleton et al., 2017). This construct can directly recruit SIN3A, a good predictor of Late gene expression responses. It also recruits Histone deacetylases (HDACs) (Urrutia, 2003), corresponding to the low H3K27ac seen at Late Up genes. For activation, dCas9-VP16(10x) was used. We have previously shown that dCas9-VP16(10x) modulates expression from enhancers and induces acetylation at targeted regions (Ginley-Hidinger et al., 2019). dCas9-VP16(10x) recruits many activating cofactors, including members of the pre-initiation complex and p300, which are associated with Early genes in the previous section.

**Figure 3.**
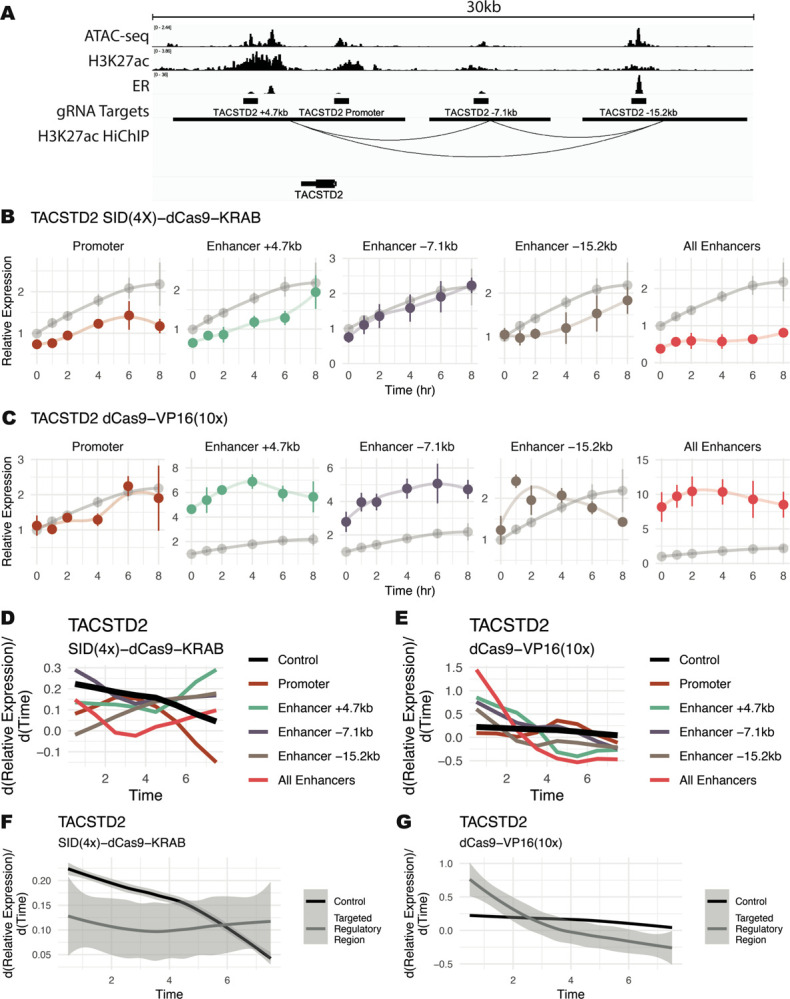
Functional manipulation of enhancer activity alters *TACSTD2* trajectory in response to estrogen. (A) ChIP-seq, ATAC-seq, and HiChIP genome browser tracks in Ishikawa cells showing targeted regulatory regions surrounding *TACSTD2*. (B-C) Expression trajectory of *TACSTD2* following estrogen induction in Ishikawa cells. (B) SID(4x)-dCas9-KRAB inhibition or (C) dCas9-VP16(10x) activation targeted to regulatory regions temporally alters the TACSTD2 response. Controls are shown in gray and lines represent loess regressions. (D-E) Differential of loess regressions from B and C show expression rates of change with regulatory regions targeted by SID(4x)-dCas9-KRAB (D) or dCas9-VP16(10x) (E). (F-G) Aggregate rates of change for all regulatory regions targeted (grey) by SID(4x)-dCas9-KRAB (F) or dCas9-VP16(10x) (G) compared to control (black).

*TACSTD2* is an E2-regulated gene that is a prognostic indicator for endometrial cancer disease-free survival (Bignotti et al., 2012), is overexpressed in some breast cancers (Shvartsur and Bonavida, 2014), and exhibits an Early Up trajectory. Targeting the enhancers of *TACSTD2*, marked by H3K27ac and ER binding (Figure 3A) with SID(4X)-dCas9-KRAB resulted in a slower, more gradual response to E2. The time for expression to reach maximal observed levels was increased when targeting 2 out of 3 individual enhancers (Figure 3B). When targeting Enhancer +4.7kb, enhancer −15.2kb, and all enhancers, lower activation rates from 0 to 4 hours were observed compared to non-targeting controls (Figure 3D). These rates were calculated usingz the differential of a loess regression. Enhancer +4.7kb and enhancer −15.2kb showed increased slope at later timepoints and similar activation levels at 8 hours compared to controls, indicating that these enhancers can regulate the timing of a response without affecting overall levels. Inhibition of the promoter also led to lower slopes across all timepoints and decreased expression levels (Figure 3B and D, dark red). Synthetic activation of the same *TACSTD2*-linked CREs led to a more rapid response when targeting each individual enhancer and the combination of all enhancers (Figure 3C), as evidenced by increased activation rates between 0 and 2 hours (Figure 3E). Again, we see that enhancer −15.2kb changes gene timing without affecting overall transcript abundance (Figure 3E, brown), and the most substantial effect on response timing is seen when targeting all enhancers in combination. These results imply that decreasing enhancer activity reduces initial activation rates while activating enhancers potentiates a gene for quicker responses to E2 (Figure 3F and G).

When targeting five putative enhancers as well as the promoter of *TGFA*, an Early Up gene, with SID(4X)-dCas9-KRAB, we again observed a more gradual expression response to E2 from 3 of 5 targeted enhancers, the promoter, and a combination of all enhancers (Figure S3A). The most substantial effects on timing were seen when targeting a distal enhancer, enhancer −62kb, or all enhancers simultaneously (Figure S3C gray and orange). On aggregate, inhibition of *TGFA* regulatory regions showed slower activation rates between early timepoints, followed by increased rates from 6 to 8 hours relative to the control trajectory (Figure S3E). These results are consistent with our *TACSTD2* findings and indicate that decreasing enhancer activity slows the transcriptional response.

To speed up a Late gene, dCas9-VP16(10x) was used to activate enhancers and the promoter of *PEG10*. 3 out of 4 enhancers, located at +45kb, +158kb, and −305kb from the transcription start site, led to a sharper increase in transcription at early timepoints than a control trajectory (Figure S3B and D). Promoter targeting did not significantly speed up the response to E2 but led to substantially higher expression levels (Figure S3B, dark red). On aggregate, we see that activation of *PEG10* enhancers led to earlier responses to E2, which later converge with control rates (Figure S3F). Overall, at these three genes, the activity of promoters and individual enhancers can control the E2 response trajectory and targeting enhancer combinations consistently modifies temporal patterns.

### An enhancer-promoter dichotomy controls gene expression noise

Genes were separated into three levels of variation to find determinants of expression noise. In scRNA-seq, low gene expression levels often have high noise due to the dropout effects of capturing RNAs from the limited amount of RNA in a single cell. Technical variation in scRNA-seq is related to mean levels (Brennecke et al., 2013). To examine mean-independent noise, we used an adjusted coefficient of variation (CV), which is calculated as the residuals of a generalized additive model (GAM) fitted to CV vs. the mean (Figure S4A). To remove any leftover mean effects, genes were labeled as high or low noise based on whether they were in the top 20% or bottom 20% of adjusted CV for ten different mean bins from the 0-hour timepoint of both cell types.

**Figure 4.**
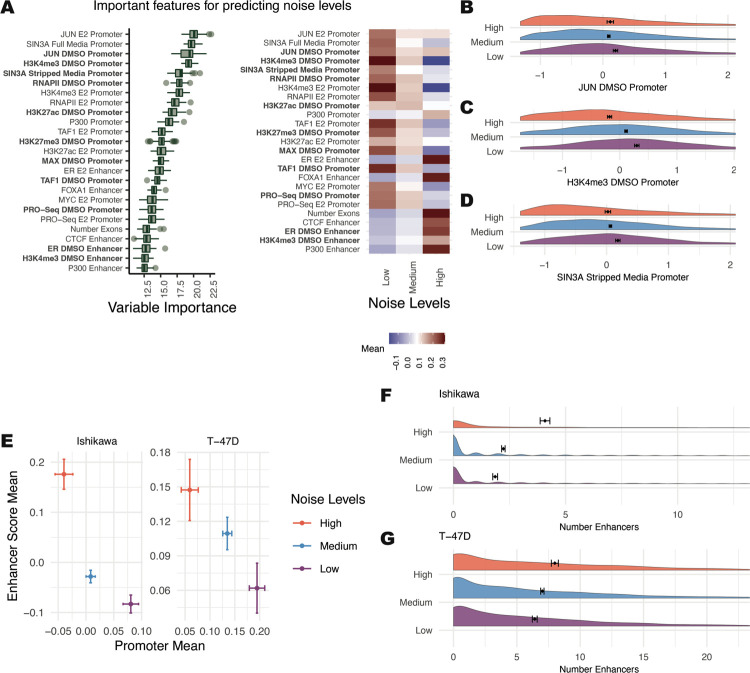
Determinants of noise levels show a balance between active promoters driving low noise levels and active enhancers driving high noise levels. (A, left) Boruta feature rankings shows features predictive of noise levels. (A, right) Average signal intensity is shown by noise group for top ranked features. Datasets shown in bold were performed in the absence of ER activation. (B-D) Distribution of signal for top ranked noise-predicting DMSO-treated features is shown with Z-scores on the x-axis. (E) Mean enhancer score signal for all Boruta confirmed features vs. mean promoter signal across all confirmed features for each noise level exhibits an inverse relationship. Error bars show 95% confidence intervals. (F-G) Distribution of enhancer counts per gene, separated by noise level, are shown for (F) Ishikawa and (G) T-47D cells.

The strongest predictors of noise levels were SIN3A and JUN at the promoter, both associated with low noise (Figure 4A–B,D). Generally, a strong promoter signal was related to low noise across features, with some exceptions, such as p300 (Figure 4A, right panel). Most enhancer features were associated with high noise, with ER and FOXA1 at enhancers being the most predictive (Figure 4A). Another feature scored as highly important was tri-methylation at histone H3 lysine 4 (H3K4me3), which is strongly associated with low amounts of noise (Figure 4C), supporting a role for H3K4me3 in controlling noise. This result is consistent with a previous study that found a relationship between H3K4me3 breadth and transcriptional consistency (Benayoun et al., 2014).

These results motivated the broader evaluation of how noise relates to promoter and enhancer activity. Analysis of the average promoter intensity across all confirmed datasets and the average enhancer score revealed an inverse relationship between promoters and enhancers (Figure 4E). Genes with high noise levels had high enhancer scores and low promoter signals. Conversely, genes with low noise levels had low enhancer scores and high promoter signals. To confirm this relationship in a third cell type, we analyzed publicly available scRNA-seq and genomic data from LNCaP cells, a prostate cancer cell line. The same association was observed between enhancer-driven gene regulation and higher noise (Figure S4B). Consistent with these findings, more enhancers connected to a gene is associated with greater noise (Figure 4F and G).

### Several features are associated with noise and either expression levels or timing

**Figure 5.**
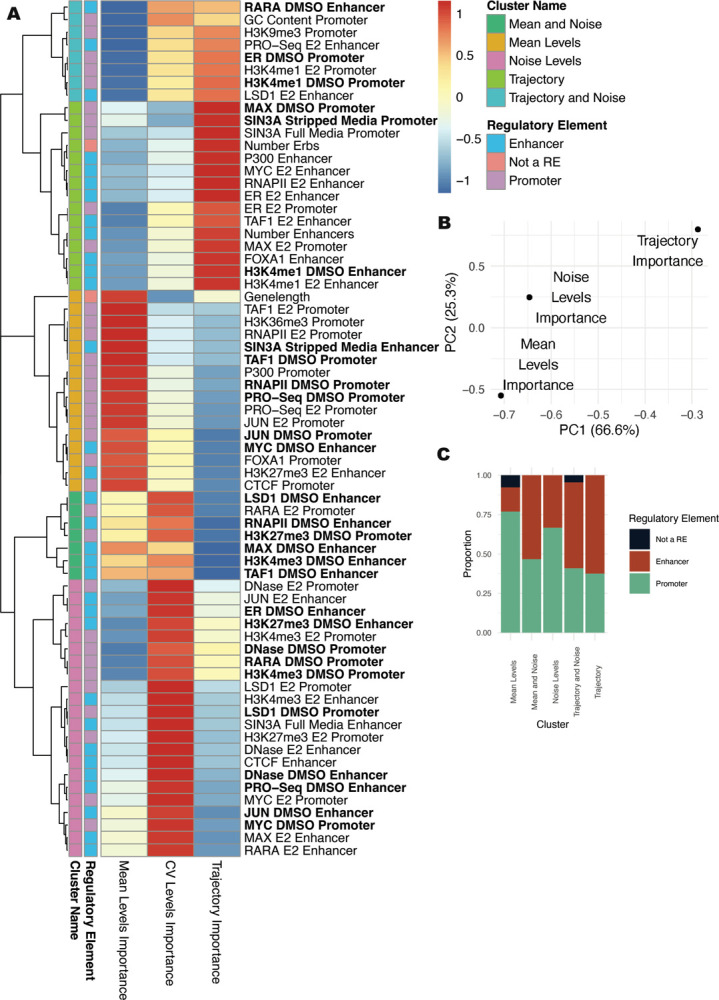
Importance comparison shows that mean and trajectory are regulated by distinct genomic features. (A) Heatmap shows importance scores from each analysis type, normalized by column, and scaled by row. Datasets shown in bold were performed in the absence of ER activation. (B) PCA plot based on importance scores show the relationship of importance scores for mean levels, noise, and trajectory. Percentages denote percent of variance explained by each principal component. (C) Proportion of features associated with enhancers, promoters, or features not associated with a specific regulatory element type, are plotted by cluster.

Levels, noise, and timing analysis showed different importance rankings for genomic features. Hierarchical clustering of these features by importance score revealed five major clusters (Figure 5A). The largest clusters consisted of features specific to each analysis. Two smaller clusters were composed of factors necessary for both mean and noise or both trajectory and noise. Notably missing were factors important for both mean levels and temporal predictions. In general, the importance score from the Boruta algorithm is more similar between noise and mean or noise and trajectory compared to mean and trajectory, as seen from the first two PCA dimensions calculated from the feature importance matrix (Figure 5B) and the correlation between importance scores (Figure S5B–D). The relationship between noise and our other analyses suggests that noise may be an intermediary between baseline levels and temporal regulation and that mean levels do not strongly influence response trajectory.

By comparing the ratio of promoter to enhancer features in each cluster, we see that mean levels are the most promoter driven. Noise and trajectory utilize promoter and enhancer features more evenly (Figure 5C). Enhancer features important for predicting mean levels are also likely to predict noise. Together, these results indicate that enhancers are more critical for noise and trajectory and that many genomic signals preferentially predict either levels, noise, or trajectory. Boruta importance scores do not capture directionality. To examine which features are associated with classification groups for each analysis, the group with the maximum signal is shown in Figure S5A. Promoter features are generally associated with high mean levels and low noise. Enhancer features are also associated with increased mean levels, but contrary to promoters, they show a higher signal for high noise levels. Enhancer scores are almost always the highest for Early Up trajectories (Figure S5A). Our results suggest that active enhancers drive high noise and rapid up-regulation in response to E2, while promoters consistently drive low noise and high mean expression.

### Co-expression of genes is based on looping, timing, and noise levels

**Figure 6.**
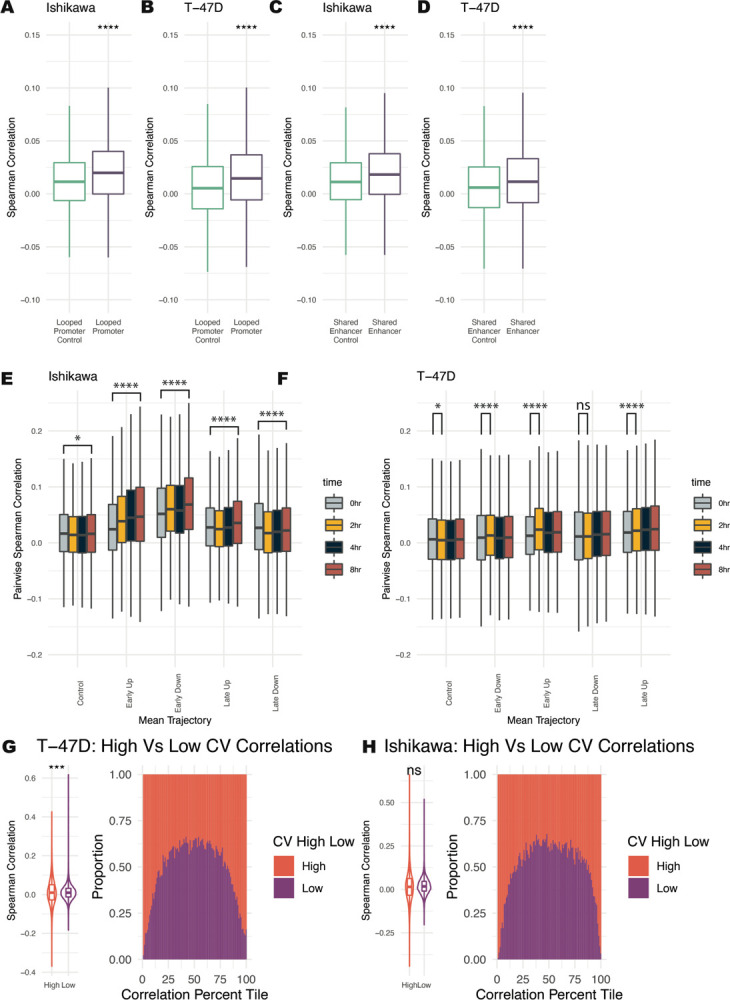
Co-expression changes are observed based on looping, trajectory, and levels of noise. (A-B) Pairs of genes with promoters that loop to one another are significantly more correlated across cells at the 0-hour timepoint than randomly selected gene pairs for Ishikawa (A) and T-47D (B). Bonferroni adjusted Wilcoxon p-values are shown with respect to control. (C-D) Pairs of genes with a shared enhancer are more correlated than randomly paired genes for Ishikawa (C) and T-47D (D). Wilcoxon p-values are shown with respect to control. (E-F) Pairwise spearman correlation for genes within different trajectories is shown. Significance values show Wilcoxon p-values of the 8-hour (E) or the 2-hour (F) timepoint with respect to the 0-hour timepoint. (G-H) Range of pairwise correlations for high noise levels is greater than the range for pairs of low noise genes in Ishikawa (G) and T-47D (H). (left panel) Distribution of Spearman pairwise correlations for genes with high and low noise. (right panel) Spearman correlations were grouped into quantiles and bars show proportion at each quantile that are pairs of low or high noise genes. Significance values for all subpanels are as follows: (* p < 0.05; ** p < 1×10–5; *** p < 1×10–10; **** p < 1×10–15).

scRNA-seq offers a unique advantage in studying the co-regulation of genes and the possible mechanisms that underlie co-regulation. Using the H3K27ac HiChIP data, we found that looping can affect co-expression in several ways. First, we found that genes whose promoters loop together correlate significantly more than groups of randomly paired control genes at the 0-hour timepoint (Figure 6A and B). Genes whose promoters both loop to a shared enhancer are significantly more correlated across single cells (Figure 6C and D). These results indicate that the 3D genome structure may be involved or associated with gene co-expression across single cells.

We next evaluated co-expression during the E2 treatment time course. Co-expression was measured using pairwise spearman correlation in single cells. In Ishikawa cells, both Early Up and Early Down genes show increasing pairwise co-expression over time (Figure 6E). Genes that respond late exhibited less change in correlation, with Late Up genes increasing correlation slightly by 8 hours and Late Down genes slightly decreasing in correlation. In T-47D cells, we see the most significant increase in co-correlation at 2 hours for Early Up and Early Down genes (Figure 6F). Late Up and Late Down genes show slight increases in correlation during the time course.

The levels of noise also change the probability of two genes being correlated. Perhaps expected, genes with high noise levels also show a broader distribution in their pairwise correlations, resulting in genes with high noise being more likely to have extremely correlated or anti-correlated expression with each other than low noise genes (Figure 6G and H). These results indicate that 3D interactions, control of temporal trajectory, and noise regulation can impact gene co-regulation.

## Discussion

To investigate the genomic underpinnings of the temporal transcriptional response to estrogen, we conducted scRNA-seq at several timepoints. scRNA-seq was able to capture more subtle changes in gene expression of highly expressed genes compared to bulk RNA-seq due to the increased statistical power. Using a feature ranking approach, we identified several features associated with E2 response timing, including SIN3A signal at the promoter of “Late Up” genes and more ER-bound enhancers regulating “Early Up” genes. In general, multiple enhancers are more predictive of “Early” gene trajectories. Functional evaluation of enhancers revealed that multiple enhancers regulate timing at each gene tested and that enhancers, in combination, are consistent regulators of expression trajectory. We conclude that an active enhancer repertoire is necessary for rapid gene responses. Active enhancers may present chromatin that is more open to ER binding. Alternatively, other TFs already present at enhancers could stabilize the binding of ER, permitting ER to activate gene expression immediately. Contrastingly, SIN3A and MAX at the promoter, known to repress gene expression together (Baudino and Cleveland, 2001), slow a gene’s response to E2, even when a gene is associated with strong ER-bound enhancers. Activation of gene expression may first require removing repressive signals at the promoter, explaining the more gradual responses. A similar mechanism has been described in an enhancer context, where inactive enhancers must first be activated by transcription factors before activation of gene expression can occur, causing more gradual gene expression changes (Ostuni *et al*., 2013).

Genomic data analysis revealed that opposing enhancer and promoter activities control expression noise. Active promoters are associated with low noise levels, whereas multiple active enhancers are associated with high noise levels. In concordance with this observation, synthetic activation of promoters drives lower noise levels at several genes (Fraser et al., 2021a). Additionally, activation of multiple enhancers causes high noise at the NF-κB locus (Wibisana *et al*., 2022). Our results support a unified model where a balance between enhancers and promoters control noise. Both intrinsic and extrinsic noise could potentially explain the observed noise distributions (Ham et al., 2021). If intrinsic noise is the driving factor, we expect promoters to cause high-frequency, near-constant transcription levels and enhancers to cause infrequent, high-amplitude bursts of expression (Raj and Van Oudenaarden, 2008). Noise caused by CREs could also be due to extrinsic noise. Promoters may lead to low noise, as fluctuations in upstream factors may be insignificant compared to activation by an ensemble of transcription factors bound to the promoter. In contrast, enhancers may drive higher noise levels by increasing sensitivity to upstream elements. Our above observation that enhancers drive rapid temporal responses further supports the theory that strong enhancers increase a gene’s sensitivity to incoming signals.

For a gene to respond quickly to a signal, it must be sensitized to incoming signals, which may inherently drive higher levels of noise. A noise-robustness tradeoff has been proposed previously when observing changes in gene expression over developmental time in *drosophila (Sigalova et al., 2020)* and in a mathematical framework that showed variations are necessary for a gene’s responsiveness (Boe et al., 2022). However, a regulatory mechanism has not been found. Our results point to multiple enhancers being a primary genomic feature associated with both high expression noise and rapid response timing. Additionally, we found that a strong promoter is likely to cause more robust gene expression but limited responsiveness. This tradeoff begs the question of whether specific genes can respond quickly to a signal without high cell-cell variation and what mechanisms may be in place to prevent noise in this case.

Co-expression analysis of gene pairs showed that co-expression properties depend on looping, timing, and noise. We found that genes with shared enhancers and looped promoters correlate more in individual cells, genes with different trajectories correlate differently over time, and genes with high noise levels are more likely to be strongly correlated or anticorrelated. These observations have considerable implications for gene regulatory networks (GRNs) in single cells, as co-expression often underlies regulatory networks. Dynamic adjustment of regulatory networks may have critical functional outcomes for a cell population (Borriello et al., 2020). For example, GRNs which confer resistance to therapeutics may occur at distinct timepoints following treatment (Zhang et al., 2019). Our results indicate that genes with high noise may lead to a broader range of implemented regulatory networks across single cells, enhancing cellular heterogeneity. Further studies into functional GRNs are warranted to determine how noise and timing affect single-cell phenotypes through the co-expression of many genes. Overall, our study shows that enhancers and promoters can play distinct roles in the timing and variation of a transcriptional response.

## Methods

### Cell culture

T-47D and Ishikawa cells were cultured in RPMI 1640 medium (Gibco) with 10% fetal bovine serum (Gibco) and 1% penicillin–streptomycin (Gibco). LNCaP cells were cultured in RPMI media with 10% FBS supplemented. Cells were incubated at 37°C with 5% CO_2_. 5 days before estrogen inductions, cells were transferred to hormone-depleted media consisting of phenol red-free RPMI (Gibco) with 10% charcoal-dextran stripped fetal bovine serum (Sigma-Aldrich) and 1% penicillin–streptomycin (Gibco).

### ChIP-seq

After 5 days in hormone-depleted media, cells were plated in 15cm dishes at approximately 60% confluency 1 day before estrogen induction. Cells were treated with vehicle (DMSO) or E2 at a final concentration of 10nM for either 1 hour for transcription factor ChIP-seqs, or 8 hours for histone marker ChIP-seqs. ChIP and library preparation was performed as previously described (Reddy et al., 2009). Antibodies used for this study were MAX (Sant Cruz sc-8011), LSD1 (abcam ab 17721), TAF1 (sc-735), c-MYC (Santa Cruz sc-40), H3K4me3 (Cell Signaling 9751S), H3K4me1 (Cell Signaling 5326S), SIN3A (produced as previously described) (Hassig et al., 1997), RARA (Santa Cruz sc-515796) and JUN (BD Biosciences 558036). Libraries were sequenced using either an Illumina HiSeq 2500 or Illumina NovaSeq 6000 as single- or paired-end 50 bp reads, then aligned to hg19 using bowtie with parameters -m 1 –t –best -q -S -l 32 -e 80 -n 2 (Langmead et al., 2009). Signal intensity was extracted from bam files using samtools view with parameter -c (Li et al., 2009). In the cases where peaks were called, peak calling was done using Macs2 with the default q-value cutoff of 0.05 and mfold ratio between 15 and 100 (Zhang et al., 2008).

### H3K27ac HiChIP

HiChIP experiments were performed as previously described (Mumbach et al., 2016) using an antibody that recognizes H3K27ac (Abcam, ab4729). Ishikawa cells were treated with either 10 nM E2 for 1 hour or DMSO as a vehicle control. HiChIP in Ishikawa cells was conducted using restriction enzyme DpnII (New England Biolabs). Crosslinked chromatin was sonicated using an EpiShear probe-in sonicator (Active Motif) with three cycles of 30 seconds at an amplitude of 40% with 30 seconds rest between cycles. HiChIP libraries were sequenced on NovaSeq 6000 as paired end 50 base pair reads to an average depth of 300–400 million read-pairs per sample.

Experiments in T-47D and LNCaP cells were conducted using restriction enzyme MboI (New England Biolabs). Crosslinked chromatin was sonicated using Covaris E220 with the settings of fill level=10, duty cycle=5, PIP=140, cycles per burst=200, time=4 mins. HiChIP libraries were sequenced on HiSeq 2500 as paired end 75 base pair reads to ~50 million read pairs per sample.

Reads were aligned to human hg19 reference genome using HiC-Pro (Servant et al., 2015). Hichipper (Lareau and Aryee, 2018) was used to perform restriction site bias-aware modeling of the output from HiC-Pro and to call interaction loops. In Ishikawa cells, DMSO and E2 treated HiChIP loops were combined to identify all possible putative enhancers. In all datasets, loops with less than 3 reads or FDR >= .05 were filtered out.

### PRO-seq

PRO-seq libraries were generated as described in Mahat et al., 2016 (Mahat et al., 2016). Briefly, Ishikawa and T-47D cells were grown in hormone-depleted RPMI for five days, then 2×10^6^ cells were plated into two 10 cm dishes per condition with RPMI lacking phenol red supplemented with 10% charcoal/dextran-stripped FBS and penicillin. Cells were treated with vehicle (DMSO) or 10 nM E2 for 45 minutes, then permeabilized for five minutes with permeabilization buffer [10 mM Tris-HCl, pH 7.4; 300 mM sucrose; 10 mM KCl; 5 mM MgCl2; 1 mM EGTA; 0.05% Tween-20; 0.1% NP40 substitute; 0.5 mM DTT, protease inhibitor cocktail ml(Roche); and SUPERaseIn RNase Inhibitor (Ambion)]. The nuclear run-on was performed by adding permeabilized cells to run-on mixture [final composition was 5 mM Tris, pH 8.0; 25 mM MgCl2; 0.5 mM DTT; 150 mM KCl; 200 μM rATP; 200 μM rGTP; 20 μM biotin-11-rCTP (Perkins Elmer); 20 μM biotin-11-rUTP (Perkins Elmer); 1 U/μL SUPERase In RNase Inhibitor (Ambion); 0.5% Sarkosyl], then incubating at 37°C for 5 minutes. RNA was extracted with Trizol LS (Ambion), fragmented with 0.2 N NaOH for 8 minutes on ice, then neutralized with 0.5 M Tris, pH 6.8, followed by buffer exchange with a P-30 column (Bio-Rad). Biotinylated RNAs were enriched with Dynabeads M280 Streptavidin (Invitrogen), then RNA was extracted with Trizol (Ambion), followed by 3′ adapter ligation using T4 RNA ligase (NEB). Biotinylated RNAs were enriched for a second time, followed by 5′ cap repair with RppH (NEB) and 5′ hydroxyl repair with PNK (NEB). The 5′ adapter was ligated with T4 RNA ligase (NEB), followed by a third biotinylated RNA enrichment. Reverse transcription was performed with the RP1 primer. Samples were PCR amplified for 13 cycles, then cleaned up with Agencourt AMPure XP beads (Beckman Coulter). Libraries were sequenced on an Illumina HiSeq 2500, generating a 50nt read. Reads were processed using cutadapt (Martin, 2011) with parameters -a TGGAATTCTCGGGTGCCAAGG --cut 7 --length 42 -m 21. Reverse complement sequences were generated using fastx_reverse_complement from the FASTX toolkit (v 0.0.13) (Hannon, 2010). Reads were then aligned to hg19 with bowtie2 (Langmead and Salzberg, 2012) in end-to-end mode, and non-uniquely aligned reads were discarded.

### scRNA-seq

Cells were treated with 10nM E2 for 0 (vehicle treated), 2, 4, and 8 hours. To mitigate technical batch effects, cells were labeled via MULTI-seq as previously described (McGinnis et al., 2019). Cells from different timepoints were mixed and then prepared according to the 10x Genomics sample prep user guide (Single Cell Suspensions from Cultured Cell Lines for Single Cell RNA Sequencing, 2017). Cells were separated into single-cell emulsions using the 10x Genomics Chromium Controller with a targeted recovery of 10,000 cells. Sequencing libraries were prepared using the 10X Genomics Next GEM Single Cell 3′ Gene Expression Library prep v3.1. Sequencing was performed on an Illumina NovaSeq 6000 with 150bp read length. Sequencing output was processed from reads to counts using the 10x Genomics Cell Ranger v3.1.0 pipeline. MULTI-seq calls were processed using the demultiplex R package (McGinnis *et al*., 2019) and mapped back to the E2 timepoints. Counts were log normalized using the Seurat v3 R package (Stuart et al., 2019), then filtered using custom cutoffs (Figure S1A and B). Genes are filtered to have a mean greater than 0.01 across all timepoints.

### scRNA-seq analysis: classification of trajectory and noise levels

Computational analysis of trajectory and noise levels were conducted using R (Team, 2013). Trajectory classification was done using a Wilcoxon test (Bauer, 1972) to find genes whose single-cell distributions significantly change at different timepoints compared to the 0hr timepoint. Genes that change significantly by 2 hours are classified as either “Early Up” or “Early Down”. Genes with changes seen at 4 or 8 hours are classified as “Late”. To select control genes with similar mean distributions to those genes that are regulated by E2, we used a stratified sampling approach to select control genes that are not significantly regulated.

Our noise metric is defined as the residuals from a generalized additive model (GAM) regression fitted to the CV vs mean for all genes. Regression was performed on log2(CV + 1) vs mean curve using the gam function from the mgcv R package with formula y ~ s(x, bs = “cs”) (Wood, 2006). Residuals were then transformed back to the original scale. Noise levels were determined using the GAM-adjusted CV. To account for the different scales of noise in different mean levels, genes were binned by mean into 3 groups by quantile. The top 20% and bottom 20% of genes in each quantile were labeled as “High” and “Low” noise, respectively.

### Feature importance analysis

Promoters were defined as 500bp regions centered on the transcriptional start site, as annotated in the RefSeq database (Pruitt et al., 2013). Enhancers were called using H3K27ac HiChIP data and H3K27ac ChIP-seq peaks. Enhancers were defined as H3K27ac peaks within HiChIP anchors that loop to the promoter. Integrated signal for each promoter and enhancer was collected from all datasets using samtools view -c (Li *et al*., 2009). Z-scores were calculated across all genes for input to feature ranking algorithms. An enhancer score was calculated to account for signal at multiple enhancers, using the formula

$$\sum_{1}^{n}{\text{log}}_{2}\left(s+1\right)$$

where n represents the number of enhancers associated with a gene and s represents the z-score of integrated genomic signal at each enhancer.

Number of enhancers was defined as the number of H3K27ac peaks which loop to the promoter, as determined by HiChIP. Number of ERBS was calculated as the number of enhancers that overlapped with ER ChIP-seq peaks. Feature ranking was performed using the Boruta package in R (Kursa and Rudnicki, 2010a) with default parameters and 100 maximum iterations. Gene length was calculated from RefSeq transcript annotations (Pruitt *et al*., 2013). An example decision tree was determined using the rpart function with parameters minbucket=50 and cp=0.007 (Breiman et al., 2017). Average enhancer score and promoter signal was calculated using “confirmed” variables from Boruta analysis. The average of Z-score signal for confirmed variables was taken for all variables associated with either the promoter or enhancer, not including number of enhancers, number of ERBS, or gene length.

### Generation of stable dCas9-VP16(10x) cell lines

Ishikawa cells were plated in 6-well plates at 60% confluency. Cells were transfected with Addgene plasmid 48227 (a gift from Rudolf Jaenisch) (Cheng et al., 2013) containing dCas9-VP16(10x) with a P2A linker and neomycin resistance gene. Fugene HD (Promega) was used for transfection at a 3:1 reagent:DNA ratio. dCas9-VP16(10x) plasmid was linearized with restriction enzyme AflII (New England Biolabs R0520S). Successful integration of the dCas9-VP16(10x) plasmid was selected for using G418 (Thermo Scientific) at a concentration of 800 μg/mL for approximately 2 weeks. Successful expression of the dCas9 plasmid was verified using qPCR for dCas9 as well as qPCR for successful activation of a control gene, *IL1RN* (Figure S3G). Cells were then maintained at a lower concentration of 400 μg/ml G418.

### gRNA design and transfection

gRNAs were designed using the Benchling gRNA design tool (Benchling, 2020). 4 gRNAs were designed per targeted region. gRNAs were cloned into plasmids as previously described (Carleton *et al*., 2017). gRNA sequence and adjacent PAM are listed in Table S1. Prior to transfection, Ishikawa cells were plated in 48-well plates at 80,000 cells/well. 24 hours after plating, gRNAs were transfected into cell using Fugene HD (Promega) at a manufacturer suggested 3:1 reagent:DNA ratio. gRNA transfection was selected for using 1 μg/mL puromycin. 8-hour E2 time courses were started roughly 24 hours after addition of puromycin.

### RNA isolation and qPCR gene expression analysis

Cells were lysed with Buffer RLT Plus (Qiagen) containing 1% beta-mercaptoethanol (Sigma). RNA was purified using the ZR-96-well Quick-RNA kit (Zymo Research). Gene expression was measured using qPCR with reagents from the Power SYBR Green RNA-to-Ct 1-step kit (Applied Biosystems), 50ng RNA per reaction, and 40 cycles on a CFX Connect light cycler (BioRad). qPCR primers are listed in Table S2. Relative expression was calculated using the ΔΔCt method with *CTCF* as a reference gene. Best fit lines were determined using the loess function in R (Cleveland et al., 2017) and formula y ~ x. Differential rates of change were calculated by determining the average slope of the loess fit for each 1-hour window.

## Supplementary Material

Supplement 1

## Data Availability

ChIP-seq, HiChIP, PRO-seq, and scRNA-seq data are available at the Gene Expression Omnibus under accession GSE227245.
