# Supplementary material for: Cis-regulatory control of transcriptional timing and noise in response to estrogen
Source: bioRxiv. 2024 Feb 14:2023.03.14.532457. Originally published 2023 Mar 15. Preprint. [Version 2] doi: 10.1101/2023.03.14.532457 (PMC10054948; doi:10.1101/2023.03.14.532457)
Supplement: Supplement 1 [file media-1.pdf]

Supplementary Information

Cis-regulatory control of transcriptional timing and noise in response to estrogen

Matthew Ginley-Hidinger[1,2], Hosiana Abewe[1,3], Kyle Osborne[1,3], Alexandra Richey[1,2], Noel Kitchen[1,3], Katelyn L. Mortenson[1,3], Erin M. Wissink[4], John Lis[4], Xiaoyang Zhang[1,3], Jason Gertz[1,2,3,*]

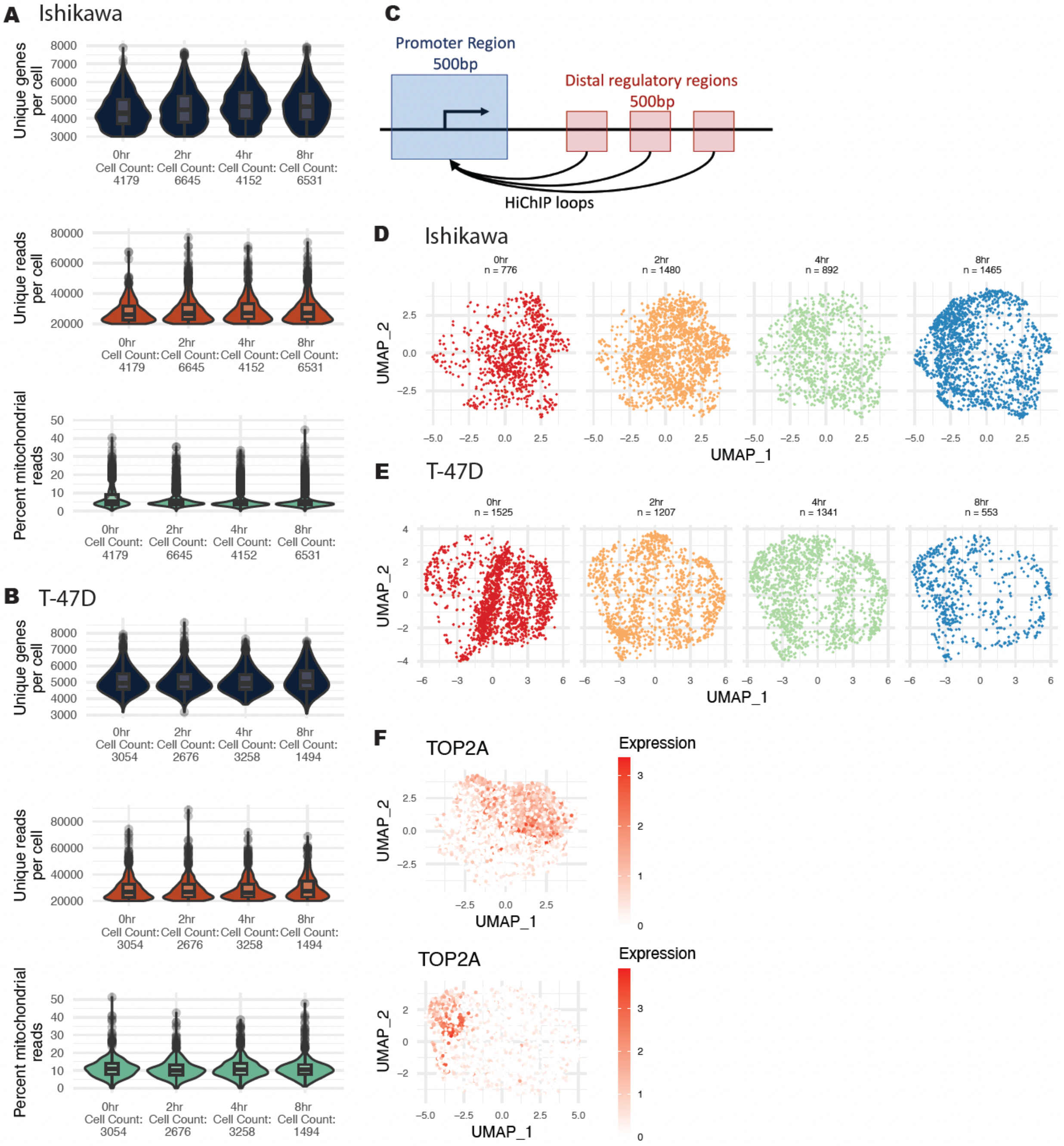

Supplementary Figure 1 (related to Figures 1 and 2). QC plots for scRNA-seq and diagram of *cis*-regulatory model. (A-B) Distribution of reads, genes, and percent mitochondrial reads per cell, separated by timepoint, are shown for (A) Ishikawa and (B) T-47D. Data shown is prior to filtering. (C) A model shows how signal intensity was collected for promoters and enhancers using HiChIP loops to identify enhancers. (D-E) UMAP dimensionality reduction plots for (D) Ishikawa and (E) T-47D show temporal progression of cells treated with E2. Each point represents a cell and timepoints are post 10 nM E2 induction. Cell numbers represent post-filtering. All cells for a cell line were used to create the original UMAP projections and the space was held constant when plotting each time point. (F) UMAP representation of cell-cycle marker gene *TOP2A,* which peaks in the G2/M phase, is shown to highlight the location of proliferating cells.

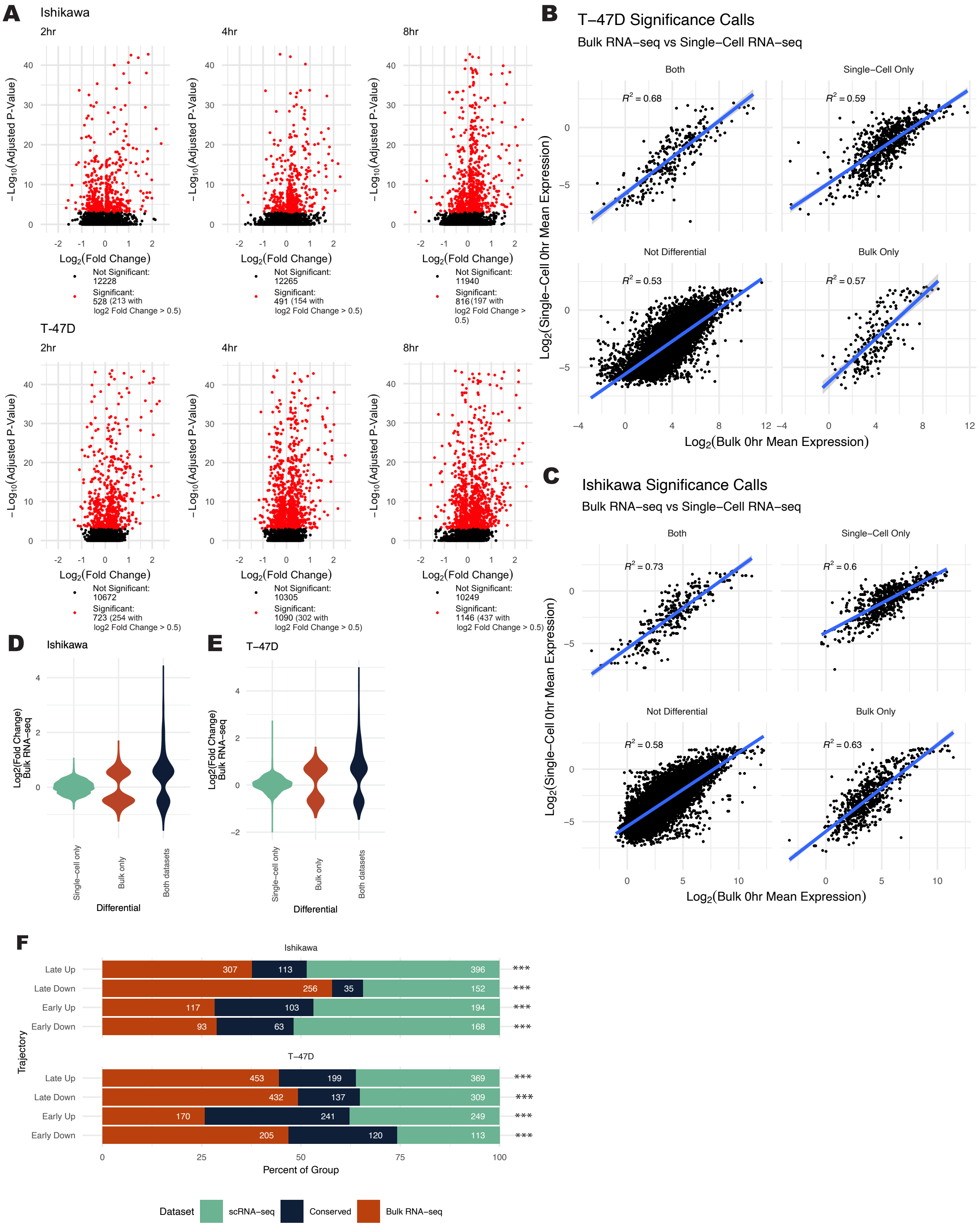
A
Ishikawa
2hr
4hr
8hr
−Log10(Adjusted P-Value)
Log2(Fold Change)
Not Significant: 12228
Significant: 528 (213 with log2 Fold Change > 0.5)
Not Significant: 12265
Significant: 491 (154 with log2 Fold Change > 0.5)
Not Significant: 11940
Significant: 816 (197 with log2 Fold Change > 0.5)
T-47D
2hr
4hr
8hr
Not Significant: 10672
Significant: 723 (254 with log2 Fold Change > 0.5)
Not Significant: 10305
Significant: 1090 (302 with log2 Fold Change > 0.5)
Not Significant: 10249
Significant: 1146 (437 with log2 Fold Change > 0.5)
B
T−47D Significance Calls
Bulk RNA−seq vs Single−Cell RNA−seq
Both
Single−Cell Only
Not Differential
Bulk Only
R2 = 0.68
R2 = 0.59
R2 = 0.53
R2 = 0.57
Log2(Single−Cell 0hr Mean Expression)
Log2(Bulk 0hr Mean Expression)
C
Ishikawa Significance Calls
Bulk RNA−seq vs Single−Cell RNA−seq
Both
Single−Cell Only
Not Differential
Bulk Only
R2 = 0.73
R2 = 0.6
R2 = 0.58
R2 = 0.63
Log2(Single−Cell 0hr Mean Expression)
Log2(Bulk 0hr Mean Expression)
D
Ishikawa
Log2(Fold Change) Bulk RNA−seq
Single−cell only
Bulk only
Both datasets
Differential
E
T−47D
Log2(Fold Change) Bulk RNA−seq
Single−cell only
Bulk only
Both datasets
Differential
F
Ishikawa
Late Up 307 113 396 ***
Late Down 256 35 152 ***
Early Up 117 103 194 ***
Early Down 93 63 168 ***
T−47D
Late Up 453 199 369 ***
Late Down 432 137 309 ***
Early Up 170 241 249 ***
Early Down 205 120 113 ***
Trajectory
Percent of Group
Dataset
scRNA−seq
Conserved
Bulk RNA−seq

Supplementary Figure 2 (related to Figure 2). Analysis of differential gene expression over time. (A) Differential gene expression -$\log_{10}$(Wilcoxon p-values) are plotted against $\log_2$(fold change) for all genes at each timepoint vs 0 hours. Significantly differential genes are highlighted in red. Data is shown for Ishikawa (top) and T-47D (bottom) cells. (B-C) Average Reads Per Million from scRNA-seq are correlated to bulk RNA-seq RPM in both Ishikawa (B) and T-47D (C) cells, shown on a log scale. Genes are separated into groups depending on whether they are called significant in bulk RNA-seq, scRNA-seq, or both. (D-E) Log_2 fold change distribution is shown for differential genes called by scRNA-seq analysis or bulk RNA-seq analysis in Ishikawa (D) and T-47D (E) cells. (F) Trajectory analysis in bulk RNA-seq vs scRNA-seq reveals a significant overlap of genes classified into each trajectory. P-values are calculated using hypergeometric tests (***: $p < 1 \times 10^{-28}$).

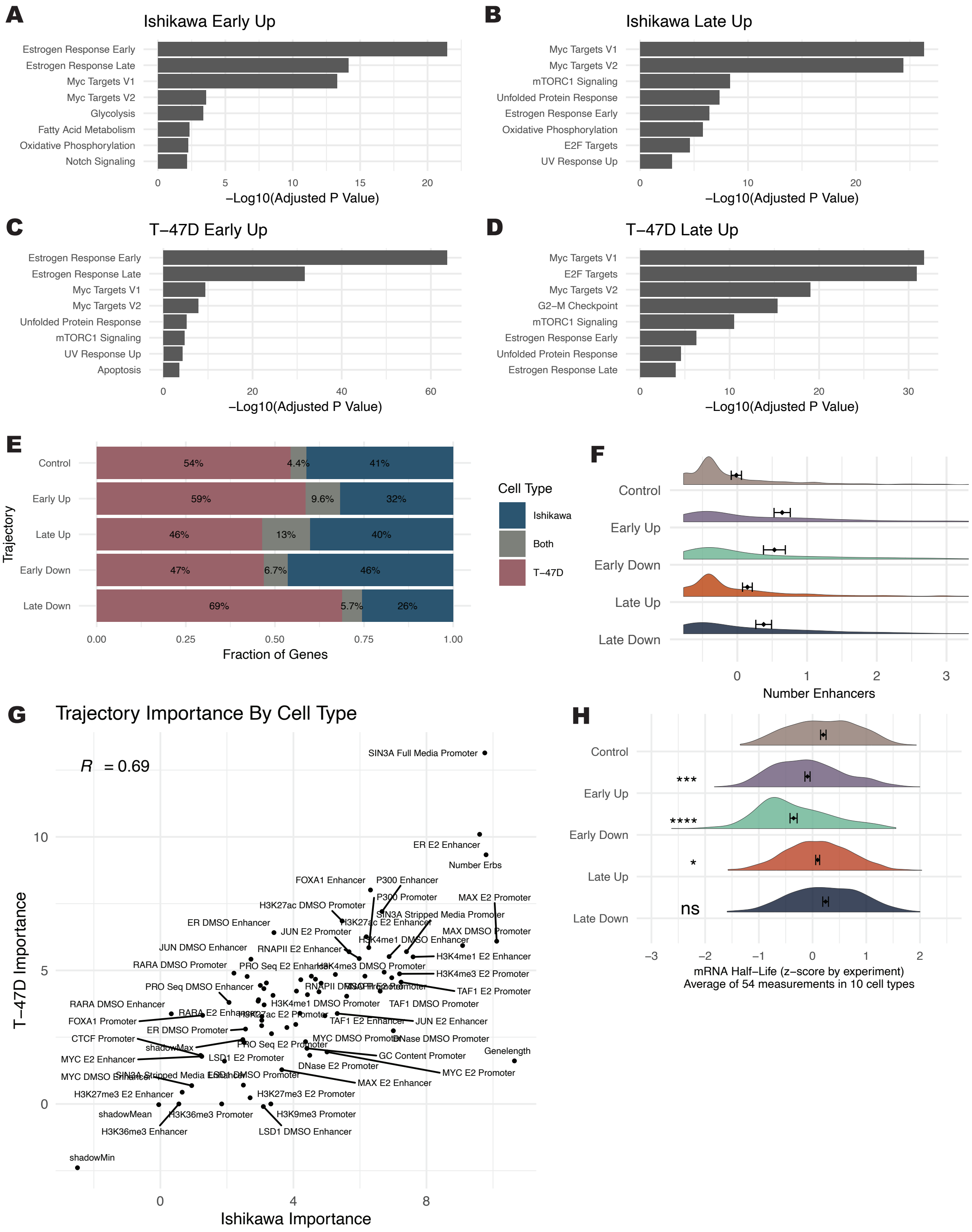

A
Ishikawa Early Up
Estrogen Response Early
Estrogen Response Late
Myc Targets V1
Myc Targets V2
Glycolysis
Fatty Acid Metabolism
Oxidative Phosphorylation
Notch Signaling
0
5
10
15
20
−Log10(Adjusted P Value)
B
Ishikawa Late Up
Myc Targets V1
Myc Targets V2
mTORC1 Signaling
Unfolded Protein Response
Estrogen Response Early
Oxidative Phosphorylation
E2F Targets
UV Response Up
0
10
20
−Log10(Adjusted P Value)
C
T−47D Early Up
Estrogen Response Early
Estrogen Response Late
Myc Targets V1
Myc Targets V2
Unfolded Protein Response
mTORC1 Signaling
UV Response Up
Apoptosis
0
20
40
60
−Log10(Adjusted P Value)
D
T−47D Late Up
Myc Targets V1
E2F Targets
Myc Targets V2
G2−M Checkpoint
mTORC1 Signaling
Estrogen Response Early
Unfolded Protein Response
Estrogen Response Late
0
10
20
30
−Log10(Adjusted P Value)
E
Trajectory
Control
54%
4.4%
41%
Early Up
59%
9.6%
32%
Late Up
46%
13%
40%
Early Down
47%
6.7%
46%
Late Down
69%
5.7%
26%
0.00
0.25
0.50
0.75
1.00
Fraction of Genes
Cell Type
Ishikawa
Both
T−47D
F
Control
Early Up
Early Down
Late Up
Late Down
0
1
2
3
Number Enhancers
G
Trajectory Importance By Cell Type
R = 0.69
SIN3A Full Media Promoter
ER E2 Enhancer
Number Erbs
FOXA1 Enhancer
P300 Enhancer
P300 Promoter
H3K27ac DMSO Promoter
MAX E2 Promoter
ER DMSO Enhancer
H3K27ac E2 Enhancer
SIN3A Stripped Media Promoter
JUN E2 Promoter
MAX DMSO Promoter
JUN DMSO Enhancer
RNAPII E2 Enhancer
H3K4me1 DMSO Enhancer
H3K4me1 E2 Enhancer
RARA DMSO Promoter
PRO Seq E2 Enhancer
H3K4me3 DMSO Promoter
H3K4me3 E2 Promoter
PRO Seq DMSO Enhancer
TAF1 E2 Promoter
RARA DMSO Enhancer
H3K4me1 DMSO Promoter
TAF1 DMSO Promoter
FOXA1 Promoter
TAF1 E2 Enhancer
JUN E2 Enhancer
ER DMSO Promoter
CTCF Promoter
MYC DMSO Promoter
DNase DMSO Promoter
shadowMax
PRO Seq E2 Promoter
Genelength
MYC E2 Enhancer
LSD1 E2 Promoter
GC Content Promoter
DNase E2 Promoter
MYC E2 Promoter
MAX E2 Enhancer
H3K27me3 E2 Enhancer
H3K27me3 E2 Promoter
shadowMean
H3K36me3 Promoter
H3K9me3 Promoter
H3K36me3 Enhancer
LSD1 DMSO Enhancer
shadowMin
T−47D Importance
0
5
10
0
4
8
Ishikawa Importance
H
Control
Early Up
***
Early Down
****
Late Up
*
Late Down
ns
−3
−2
−1
0
1
2
mRNA Half−Life (z−score by experiment)
Average of 54 measurements in 10 cell types

Supplementary Figure 3 (related to Figure 2). (A-D) Gene set enrichment of Early Up and Late Up genes for MSigDB Hallmark 2020 is shown for Ishikawa (A-B) and T-47D (C-D) cells. P-values are calculated using EnrichR. (E) Proportion of overlap between genes of each trajectory classification between cell types is shown. (F) Distribution in number of enhancers (z-score) by trajectory is plotted. Data is combined from both Ishikawa and T-47D cells. (G) The scatterplot shows median Boruta importance values for classifying trajectory in T-47D (y-axis) vs Ishikawa (x-axis). Boruta analysis was run separately in each cell type. Each data point represents a genomic feature. The correlation value was calculated using Pearson correlation. (H) mRNA half-life values from across 54 human samples were converted to Z-scores within each experiment. An average value was plotted for each gene, classified into trajectories based on the timing of the transcriptional response to E2. p-values are calculated using a Kolmogorov-Smirnov Test vs the Control group (ns > 0.05, * < 0.05, ** < 1e-5, *** < 1e-10, **** < 1e-15).

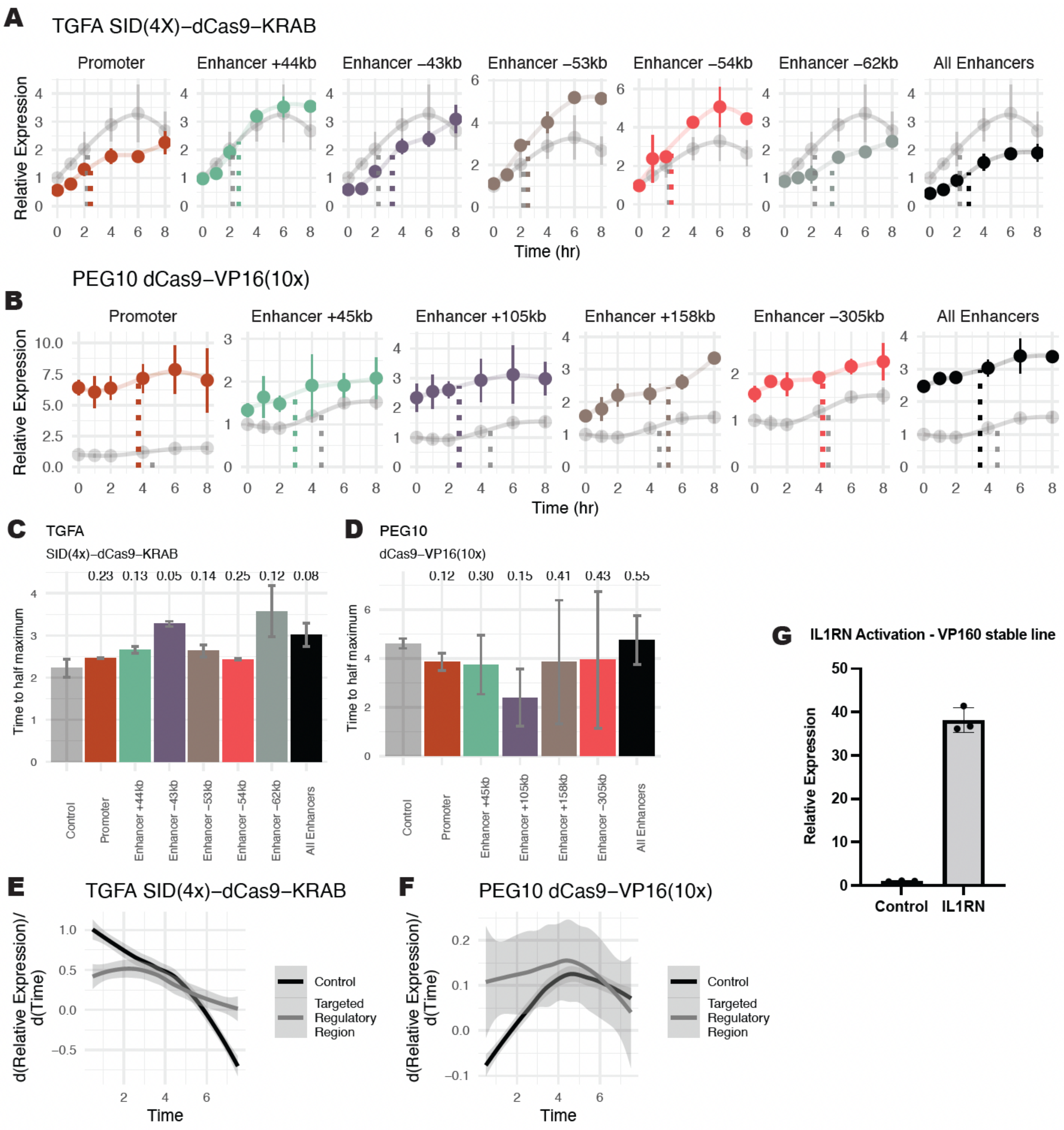
A
TGFA SID(4X)−dCas9−KRAB
Promoter
Enhancer +44kb
Enhancer −43kb
Enhancer −53kb
Enhancer −54kb
Enhancer −62kb
All Enhancers
Relative Expression
Time (hr)
B
PEG10 dCas9−VP16(10x)
Promoter
Enhancer +45kb
Enhancer +105kb
Enhancer +158kb
Enhancer −305kb
All Enhancers
Relative Expression
Time (hr)
C
TGFA
SID(4x)−dCas9−KRAB
0.23 0.13 0.05 0.14 0.25 0.12 0.08
Time to half maximum
Control
Promoter
Enhancer +44kb
Enhancer −43kb
Enhancer −53kb
Enhancer −54kb
Enhancer −62kb
All Enhancers
D
PEG10
dCas9−VP16(10x)
0.12 0.30 0.15 0.41 0.43 0.55
Time to half maximum
Control
Promoter
Enhancer +45kb
Enhancer +105kb
Enhancer +158kb
Enhancer −305kb
All Enhancers
G
IL1RN Activation - VP160 stable line
Relative Expression
Control
IL1RN
E
TGFA SID(4x)−dCas9−KRAB
d(Relative Expression)/d(Time)
Time
Control
Targeted Regulatory Region
F
PEG10 dCas9−VP16(10x)
d(Relative Expression)/d(Time)
Time
Control
Targeted Regulatory Region

Supplementary Figure 4 (related to Figure 3). Synthetic transcription factors targeted to regulatory regions alter trajectories of *TGFA* and *PEG10*. (A) Expression trajectory of *TGFA* following E2 induction in Ishikawa cells is shown. Regulatory regions are targeted with SID(4x)-dCas9-KRAB. Data from cells with *IL1RN* promoter targeting (control) are shown in gray. Dotted lines represent the time to half maximal expression for each trajectory. Expression is relative to the 0-hour timepoint in the control. (B) *PEG10* gene expression trajectories in Ishikawa cells following E2 inductions are modulated by dCas9-VP16(10x) targeted to regulatory regions. Dotted lines represent the time to half maximal expression for each trajectory. Expression is relative to the 0-hour timepoint in the control. (C-D) Bar plot shows time to half maximal expression for each targeted regulatory region. Error bars represent the SEM (n=2) and p-values (one-sided t-test) are reported above each bar. (E-F) Aggregate differential of loess regressions from panels A and B show expression slope when CREs near *TGFA* are targeted by SID(4x)-dCas9-KRAB (E) or *PEG10* CREs are targeted by dCas9-VP16(10x) (F). Slopes are aggregated targeted (grey) compared to control (black) and the shaded region represents 95% confidence interval. (G) Validation of stable dCas9-VP16(10x) expressing Ishikawa cell line shows a control gene, *IL1RN,* being activated to previously observed levels (Ginley-Hidinger et al., 2019).

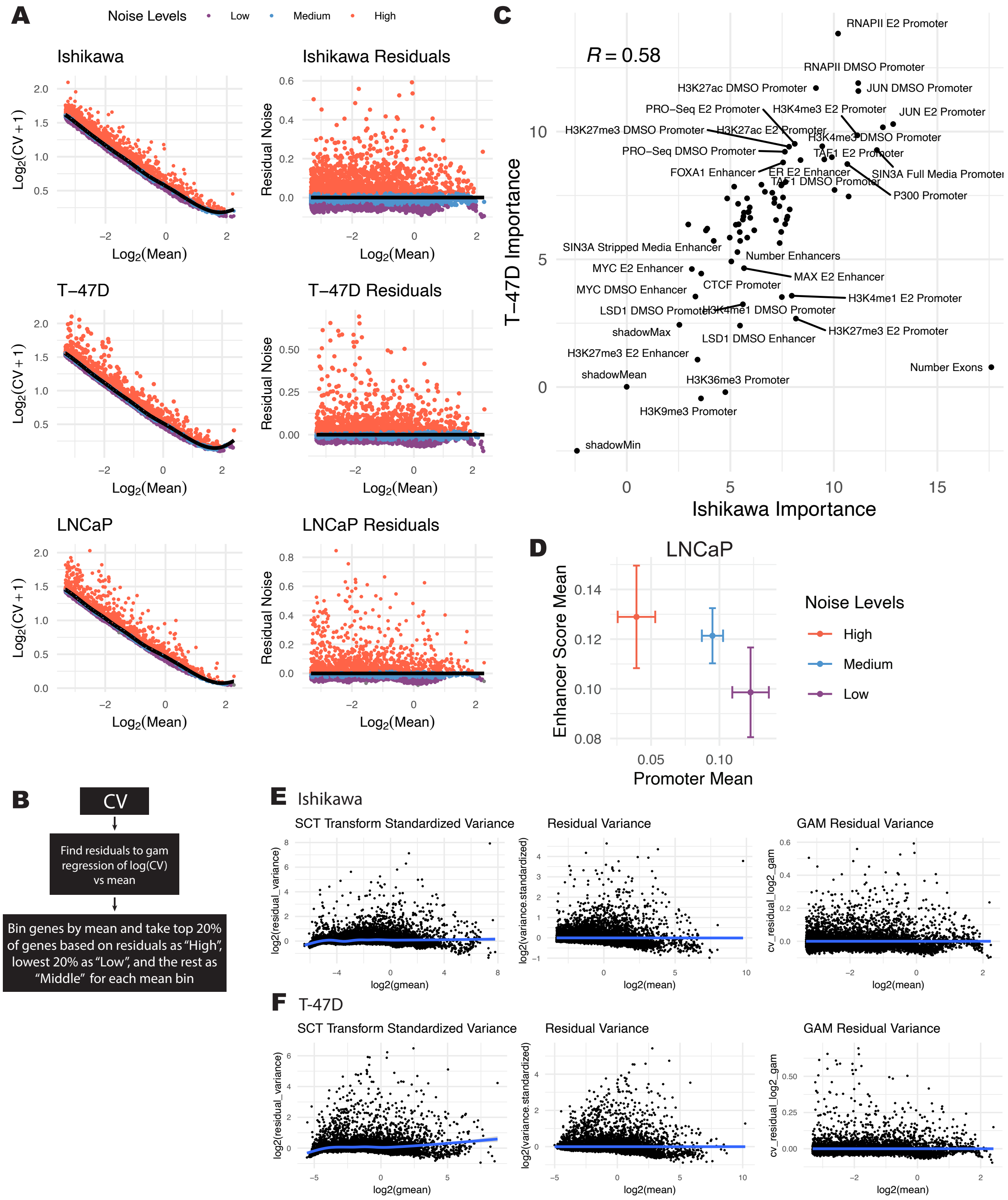
A
Noise Levels
Low
Medium
High
Ishikawa
Ishikawa Residuals
T−47D
T−47D Residuals
LNCaP
LNCaP Residuals
Log2(CV + 1)
Residual Noise
Log2(Mean)
B
CV
Find residuals to gam regression of log(CV) vs mean
Bin genes by mean and take top 20% of genes based on residuals as "High", lowest 20% as "Low", and the rest as "Middle" for each mean bin
C
R = 0.58
RNAPII E2 Promoter
RNAPII DMSO Promoter
H3K27ac DMSO Promoter
JUN DMSO Promoter
PRO−Seq E2 Promoter
H3K4me3 E2 Promoter
JUN E2 Promoter
H3K27me3 DMSO Promoter
H3K27ac E2 Promoter
H3K4me3 DMSO Promoter
PRO−Seq DMSO Promoter
TAF1 E2 Promoter
FOXA1 Enhancer
ER E2 Enhancer
SIN3A Full Media Promoter
TAF1 DMSO Promoter
P300 Promoter
SIN3A Stripped Media Enhancer
Number Enhancers
MYC E2 Enhancer
MAX E2 Enhancer
CTCF Promoter
MYC DMSO Enhancer
H3K4me1 E2 Promoter
LSD1 DMSO Promoter
H3K4me1 DMSO Promoter
shadowMax
H3K27me3 E2 Promoter
LSD1 DMSO Enhancer
H3K27me3 E2 Enhancer
Number Exons
shadowMean
H3K36me3 Promoter
H3K9me3 Promoter
shadowMin
T−47D Importance
Ishikawa Importance
D
LNCaP
Enhancer Score Mean
Promoter Mean
Noise Levels
High
Medium
Low
E
Ishikawa
SCT Transform Standardized Variance
Residual Variance
GAM Residual Variance
log2(residual_variance)
log2(variance.standardized)
cv_residual_log2_gam
log2(gmean)
log2(mean)
F
T-47D
SCT Transform Standardized Variance
Residual Variance
GAM Residual Variance
log2(residual_variance)
log2(variance.standardized)
cv_residual_log2_gam
log2(gmean)
log2(mean)

Supplementary Figure 5 (related to Figure 4). Strong enhancers and weak promoters are associated with high levels of noise. (A) Calculated coefficient of variation (CV) is shown before (left panel) and after (right panel) adjustment using a generalized additive model regression fit for each cell type. Axes are on a log scale. Colors represent classified noise levels. (B) A flowchart depicts the regularization strategy to remove mean effects from the measured variation and to classify genes into noise levels. (C) Boruta importance scores for classifying genes into noise categories are shown for independent analyses in T-47D (y-axis) and Ishikawa (x-axis) cells. Correlation value was calculated using Pearson correlation. (D) Enhancer score averages vs promoter score averages are shown for noise levels in LNCaP cells. Averages are based on confirmed variables from a Boruta analysis. Axes do not represent full range. For a list of genomic data used in LNCaP cells see Table S3. (E-F) A comparison of noise regularization strategies in (E) Ishikawa and (F) T-47D cells is shown. SCT transform standardized variance and residual variance were calculated using the Seurat package in R. Blue line shows a loess best fit.

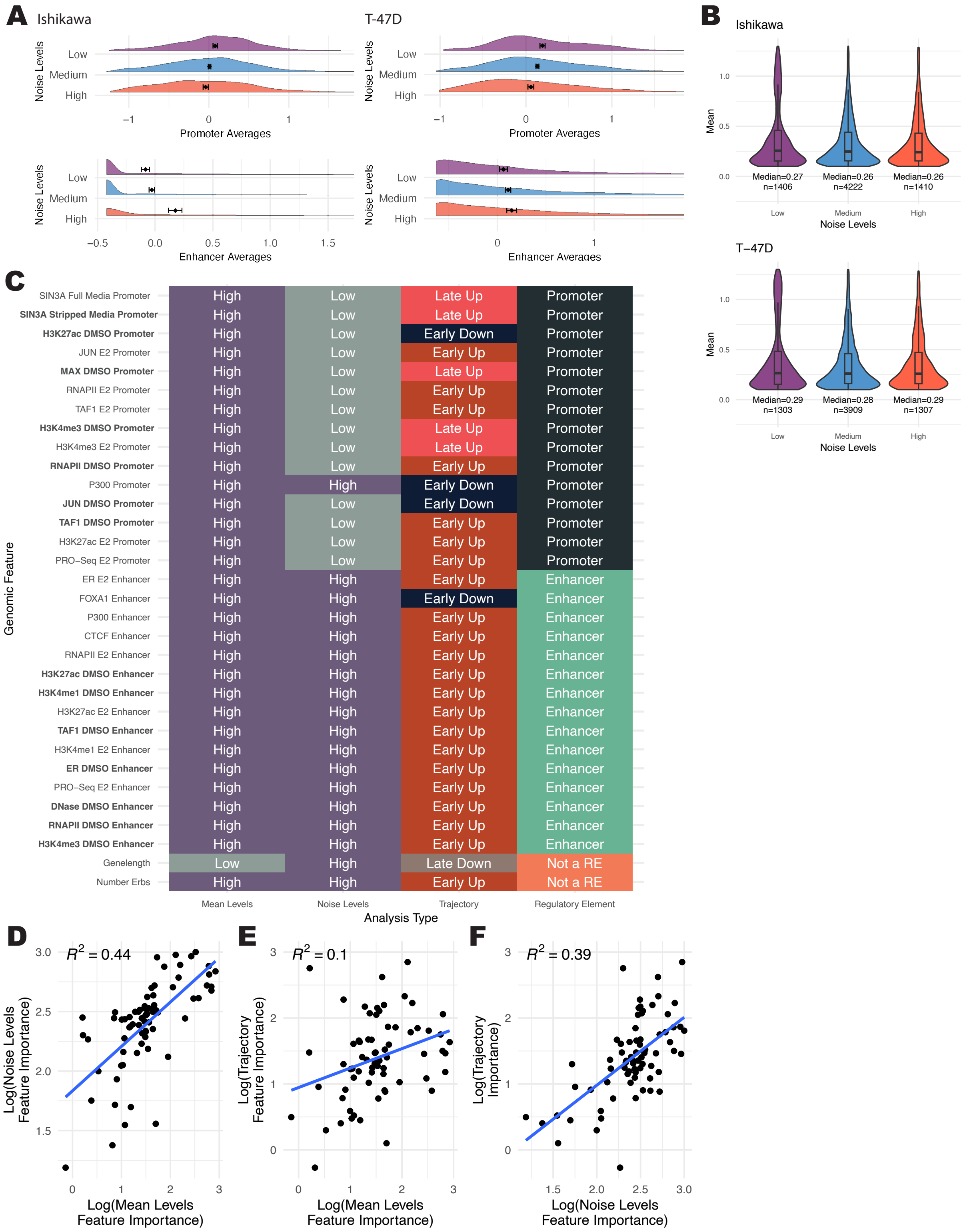
A
Ishikawa
T-47D
Noise Levels
Low
Medium
High
Promoter Averages
Enhancer Averages
B
Ishikawa
Mean
Median=0.27
n=1406
Median=0.26
n=4222
Median=0.26
n=1410
Noise Levels
T−47D
Median=0.29
n=1303
Median=0.28
n=3909
Median=0.29
n=1307
C
Genomic Feature
SIN3A Full Media Promoter
SIN3A Stripped Media Promoter
H3K27ac DMSO Promoter
JUN E2 Promoter
MAX DMSO Promoter
RNAPII E2 Promoter
TAF1 E2 Promoter
H3K4me3 DMSO Promoter
H3K4me3 E2 Promoter
RNAPII DMSO Promoter
P300 Promoter
JUN DMSO Promoter
TAF1 DMSO Promoter
H3K27ac E2 Promoter
PRO−Seq E2 Promoter
ER E2 Enhancer
FOXA1 Enhancer
P300 Enhancer
CTCF Enhancer
RNAPII E2 Enhancer
H3K27ac DMSO Enhancer
H3K4me1 DMSO Enhancer
H3K27ac E2 Enhancer
TAF1 DMSO Enhancer
H3K4me1 E2 Enhancer
ER DMSO Enhancer
PRO−Seq E2 Enhancer
DNase DMSO Enhancer
RNAPII DMSO Enhancer
H3K4me3 DMSO Enhancer
Genelength
Number Erbs
Mean Levels
Noise Levels
Trajectory
Regulatory Element
Analysis Type
High
Low
Late Up
Early Up
Early Down
Late Down
Promoter
Enhancer
Not a RE
D
$R^2 = 0.44$
Log(Noise Levels Feature Importance)
Log(Mean Levels Feature Importance)
E
$R^2 = 0.1$
Log(Trajectory Feature Importance)
Log(Mean Levels Feature Importance)
F
$R^2 = 0.39$
Log(Trajectory Importance)
Log(Noise Levels Feature Importance)

Supplementary Figure 6 (related to Figure 5). Promoters and enhancers underlie different relationships between mean, noise, and trajectory. (A) The full distribution of promoter (top) and enhancer (bottom) in Ishikawa (left) and T-47D (right) across noise levels is shown. (B) Graph depicts mean levels across different noise levels for Ishikawa (top) and T-47D (bottom). (C) Top 15 most important features for each regulatory element type (promoter or enhancer) are shown, determined by average rank across all three analysis types. For each analysis, the classification group with the highest signal is displayed. Datasets shown in bold were performed in the absence of ER activation. (D-F) Pairwise correlation plots show the relationship between importance scores for the three analysis types. Each point represents a genomic feature. R^2 values were calculated using Spearman correlation.

Table S1. gRNA sequences

gRNA Name	Sequence	PAM
PEG10 Promoter gRNA 1	GCCTTATATAGGTTGCACCG	AGG
PEG10 Promoter gRNA 2	CGCGTGGTGAGTATGCGGTG	AGG
PEG10 Promoter gRNA 3	GGAGTACGGGATTACCGAGG	GGG
PEG10 Promoter gRNA 4	TTAGTGGCCTGCGCGCGACG	TGG
PEG10 Enhancer +105kb gRNA 1	CATCTCCAGTTCAAACAGTG	AGG
PEG10 Enhancer +105kb gRNA 2	AAGCCCAGGTATATTAATGG	AGG
PEG10 Enhancer +105kb gRNA 3	TGACATAAACTAGCTGAGCA	GGG
PEG10 Enhancer +105kb gRNA 4	ACTTGAGCTTGGATCCAGCT	GGG
PEG10 Enhancer +158kb gRNA 1	GAGAGTTCCACTCTCGTGAA	TGG
PEG10 Enhancer +158kb gRNA 2	GACAGAGCAAGACTCCGTAA	AGG
PEG10 Enhancer +158kb gRNA 3	ATCTGCTGGAACCTTGATCA	TGG
PEG10 Enhancer +158kb gRNA 4	GAGGAGAGCCTCTTGAACCT	GGG
PEG10 Enhancer +45kb gRNA 1	GGAATATGCTCATAACCACA	AGG
PEG10 Enhancer +45kb gRNA 2	CTCTTAATCTCCCTTCACCA	AGG
PEG10 Enhancer +45kb gRNA 3	TCCTATTTCCTGTTGCCCTG	GGG
PEG10 Enhancer +45kb gRNA 4	CCTAATTCCAGGCTCACCAC	AGG
PEG10 Enhancer +-305kb gRNA 1	GGCATACTGGAATAAACATG	AGG
PEG10 Enhancer +-305kb gRNA 2	TGAGAGACTCACTTGTCACT	AGG
PEG10 Enhancer +-305kb gRNA 3	ACACTTCTAGAAGGCCCACT	AGG
PEG10 Enhancer +-305kb gRNA 4	TAAAGAAGGTATAAGATCCA	TGG
TACSTD2 Promoter gRNA 1	GGTAGAGTATAAGAGCCGGA	GGG
TACSTD2 Promoter gRNA 2	TGTTCTGATCCTATCGCGGG	CGG
TACSTD2 Promoter gRNA 3	GAGAACCGATACTCACCTGT	AGG
TACSTD2 Promoter gRNA 4	TAACACCAGTCCTGACAGGT	AGG
TACSTD2 Enhancer +4.7kb gRNA 1	CATCAGGAGACTAACCAGGG	AGG
TACSTD2 Enhancer +4.7kb gRNA 2	GAAGACACTGAAGCTCATAG	AGG
TACSTD2 Enhancer +4.7kb gRNA 3	AAGCCCTGGTGACCTACACA	AGG
TACSTD2 Enhancer +4.7kb gRNA 4	CAGTGAACCAGTGAACTGTA	AGG
TACSTD2 Enhancer -7.1kb gRNA 1	TGTAAGGGCTTTACACTCTG	GGG
TACSTD2 Enhancer -7.1kb gRNA 2	AGCAGAACTGTACACATCAC	AGG
TACSTD2 Enhancer -7.1kb gRNA 3	GCACCGTGACTCATCAGTGT	GGG
TACSTD2 Enhancer -7.1kb gRNA 4	GATGTGTGATGCCCTGTTAA	GGG
TACSTD2 Enhancer -15.2kb gRNA 1	TGGATTCAAGATCGAAACCC	TGG
TACSTD2 Enhancer -15.2kb gRNA 2	TGACTTCATCAGATGGAAAG	TGG
TACSTD2 Enhancer -15.2kb gRNA 3	GACATGAACGAGTCCTTGGA	AGG
TACSTD2 Enhancer -15.2kb gRNA 4	TGGGCAGGCTGATTAATGAG	GGG
TGFA Enhancer +44kb gRNA 1	TGAGTACCCAGCTCATGCA	AGG

TGFA Enhancer +44kb gRNA 2	GGGCATTACTCAGGCGCCC	GGG
TGFA Enhancer +44kb gRNA 3	CCCTGGTTAAGAAGTTGTT	GGG
TGFA Enhancer +44kb gRNA 4	GACAGGTGTTCTAAGCTAT	AGG
TGFA Promoter gRNA 1	CCCCGGTCGCCGAGTGGCG	AGG
TGFA Promoter gRNA 2	GAGCGCACCGAGCAGGGCG	CGG
TGFA Promoter gRNA 3	GCACGGCGCTGGCAGTCGG	GGG
TGFA Promoter gRNA 4	TAGGTAACGGGTGCCCGGG	CGG
TGFA Enhancer -43kb gRNA 1	GGAAGCGGGATGCAGACCT	GGG
TGFA Enhancer -43kb gRNA 2	CAAGACATCAGGCTTCCTG	GGG
TGFA Enhancer -43kb gRNA 3	GTTCACCACAGCGTGTCAA	GGG
TGFA Enhancer -43kb gRNA 4	CCAGGATCTGCCACCCCTG	TGG
TGFA Enhancer -53kb gRNA 1	CACCGAATCAACTGAGGTC	AGG
TGFA Enhancer -53kb gRNA 2	GGAGTTCCTACAATCACAG	AGG
TGFA Enhancer -53kb gRNA 3	AAGACAACGGATGTGTCCG	TGG
TGFA Enhancer -53kb gRNA 4	AGAAGCCCGAACCCAGAGA	AGG
TGFA Enhancer -54kb gRNA 1	GAACGGGGACTTGAGGTAT	TGG
TGFA Enhancer -54kb gRNA 2	AGCATCACGCTGAGAATGG	GGG
TGFA Enhancer -54kb gRNA 3	GGACATCCACTTGAAAAGC	TGG
TGFA Enhancer -54kb gRNA 4	TAAAATGAGTGCTCTAGGC	AGG
TGFA Enhancer -62kb gRNA 1	AGGGCTACCTAACTGCAAT	AGG
TGFA Enhancer -62kb gRNA 2	ATTGTCTGAACTCCGGATG	TGG
TGFA Enhancer -62kb gRNA 3	TCAGGGAGTGTTGCAACCT	TGG
TGFA Enhancer -62kb gRNA 4	GTATCACAGCGAAGCGGGT	GGG

Table S2. qPCR primers

Primer name	Sequence
TACSTD2_F	ACAACGATGGCCTCTACGAC
TACSTD2_R	GTCCAGGTCTGAGTGGTTGAA
PEG10_F	GAGCACCAGGGATTTCTCAGT
PEG10_R	GGTAGTTGTGCATCAGGTAGTG
TGFA_F	AGGTCCGAAAACACTGTGAGT
TGFA_R	AGCAAGCGGTTCTTCCCTTC
IL1RN_F	GGAATCCATGGAGGGAAGAT
IL1RN_R	TGTTCTCGCTCAGGTCAGTG
dCas9_F	GTGACCGAGGGAATGAGAAA
dCas9_R	AGCTGCTTCACGGTCACTTT

Table S3. Genomic Source Information

* - indicates experiments conducted for this study. See Data Availability.

Cell Line	Genomic Feature	Reference	Accession
Ishikawa	CTCF	(ENCODE, 2012)	GSE32465
Ishikawa	Dnase	(ENCODE, 2012)	GSE32970
Ishikawa	ER	(ENCODE, 2012; Rodriguez et al., 2020)	GSE32465; GSE129803
Ishikawa	FOXA1	(ENCODE, 2012)	GSE32465
Ishikawa	GC Content	(Kent et al., 2002)	
Ishikawa	Gene length	(Kent *et al.*, 2002)	
Ishikawa	H3K27ac	(Vahrenkamp et al., 2018)	GSE109893
Ishikawa	H3K27me3	*	
Ishikawa	H3K36me3	*	
Ishikawa	H3K4me1	*	
Ishikawa	H3K4me3	*	
Ishikawa	H3K9me3	(Carleton et al., 2017)	GSE99906
Ishikawa	JUN	*	
Ishikawa	LSD1	*	
Ishikawa	MAX	*	
Ishikawa	MYC	*	
Ishikawa	Number Enhancers	*	
Ishikawa	Number ERBS	*	
Ishikawa	p300	(ENCODE, 2012)	GSE32465
Ishikawa	PRO-seq	*	
Ishikawa	RARA	*	
Ishikawa	RNAPII	(Carleton *et al.*, 2017; ENCODE, 2012)	GSE32465; GSE99906

Ishikawa	SIN3A	*	
Ishikawa	TAF1	*	
Ishikawa	scRNA-seq	*	
Ishikawa	HiChIP	*	
T-47D	CTCF	(ENCODE, 2012)	GSE32465
T-47D	Dnase	(ENCODE, 2012)	GSE32970
T-47D	ER	(ENCODE, 2012)	GSE32465
T-47D	FOXA1	(ENCODE, 2012)	GSE32465
T-47D	GC Content	(Kent *et al.*, 2002)	
T-47D	Gene length	(Kent *et al.*, 2002)	
T-47D	H3K27ac	(Carleton et al., 2020; Zhang et al., 2016b)	GSE63109; GSE147141
T-47D	H3K27me3	*	
T-47D	H3K36me3	(Zhang et al., 2016a)	GSE63109
T-47D	H3K4me1	*	
T-47D	H3K4me3	*	
T-47D	H3K9me3	(Zhang *et al.*, 2016a)	GSE63109
T-47D	JUN	*	
T-47D	LSD1	*	
T-47D	MAX	*	
T-47D	MYC	*	
T-47D	Number Enhancers	*	
T-47D	Number ERBS	*	
T-47D	p300	(ENCODE, 2012)	GSE32465
T-47D	PRO-seq	*	
T-47D	RARA	*	
T-47D	RNAPII	*	
T-47D	SIN3A	*	
T-47D	TAF1	*	
T-47D	scRNA-seq	*	
T-47D	HiChIP	*	
LNCaP	H3ac	(Takayama et al., 2015; Yamamoto et al., 2019)	GSE62492
LNCaP	AR	(Takayama *et al.*, 2015; Yamamoto *et al.*, 2019)	GSE62492
LNCaP	CHD1	(Metzger et al., 2016)	GSE64530
LNCaP	ETV1	(Chen et al., 2013)	GSE47120
LNCaP	FOXP1	(Takayama *et al.*, 2015; Yamamoto *et al.*, 2019)	GSE62492
LNCaP	H3K27ac	(Taberlay et al., 2016)	GSE73785

LNCaP	H3K27me3	(Takayama *et al.*, 2015; Yamamoto *et al.*, 2019)	GSE62492
LNCaP	H3K4me1	(Takayama *et al.*, 2015; Yamamoto *et al.*, 2019)	GSE62492
LNCaP	H3K4me3	(Takayama *et al.*, 2015; Yamamoto *et al.*, 2019)	GSE62492
LNCaP	LSD1	(Metzger *et al.*, 2016)	GSE64530
LNCaP	RUNX1	(Takayama *et al.*, 2015; Yamamoto *et al.*, 2019)	GSE62492
LNCaP	scRNA-seq	(Taavitsainen et al., 2021)	GSE168668
LNCaP	HiChIP	*	

Supplemental References

Carleton, J.B., Berrett, K.C., and Gertz, J. (2017). Multiplex Enhancer Interference Reveals Collaborative Control of Gene Regulation by Estrogen Receptor α-Bound Enhancers. Cell Syst *5*, 333-344.e335. 10.1016/j.cels.2017.08.011.

Carleton, J.B., Ginley-Hidinger, M., Berrett, K.C., Layer, R.M., Quinlan, A.R., and Gertz, J. (2020). Regulatory sharing between estrogen receptor α bound enhancers. Nucleic Acids Research *48*, 6597-6610. 10.1093/nar/gkaa454.

Chen, Y., Chi, P., Rockowitz, S., Iaquinta, P.J., Shamu, T., Shukla, S., Gao, D., Sirota, I., Carver, B.S., and Wongvipat, J. (2013). ETS factors reprogram the androgen receptor cistrome and prime prostate tumorigenesis in response to PTEN loss. Nature medicine *19*, 1023-1029.

ENCODE, C. (2012). An integrated encyclopedia of DNA elements in the human genome. Nature *489*, 57-74. 10.1038/nature11247.

Ginley-Hidinger, M., Carleton, J.B., Rodriguez, A.C., Berrett, K.C., and Gertz, J. (2019). Sufficiency analysis of estrogen responsive enhancers using synthetic activators. Life Science Alliance *2*, e201900497. 10.26508/lsa.201900497.

Kent, W.J., Sugnet, C.W., Furey, T.S., Roskin, K.M., Pringle, T.H., Zahler, A.M., and Haussler, D. (2002). The human genome browser at UCSC. Genome research *12*, 996-1006.

Metzger, E., Willmann, D., McMillan, J., Forne, I., Metzger, P., Gerhardt, S., Petroll, K., Von Maessenhausen, A., Urban, S., and Schott, A.-K. (2016). Assembly of methylated KDM1A and CHD1 drives androgen receptor–dependent transcription and translocation. Nature structural & molecular biology *23*, 132-139.

Rodriguez, A.C., Vahrenkamp, J.M., Berrett, K.C., Clark, K.A., Guillen, K.P., Scherer, S.D., Yang, C.-H., Welm, B.E., Janát-Amsbury, M.M., and Graves, B.J. (2020). ETV4 is necessary for estrogen signaling and growth in endometrial cancer cells. Cancer research *80*, 1234-1245.

Taavitsainen, S., Engedal, N., Cao, S., Handle, F., Erickson, A., Prekovic, S., Wetterskog, D., Tolonen, T., Vuorinen, E.M., Kiviaho, A., et al. (2021). Single-cell ATAC and RNA sequencing reveal pre-existing and persistent cells associated with prostate cancer relapse. Nat Commun *12*, 5307. 10.1038/s41467-021-25624-1.

Taberlay, P.C., Achinger-Kawecka, J., Lun, A.T., Buske, F.A., Sabir, K., Gould, C.M., Zotenko, E., Bert, S.A., Giles, K.A., and Bauer, D.C. (2016). Three-dimensional disorganization of the cancer genome occurs coincident with long-range genetic and epigenetic alterations. Genome research *26*, 719-731.

Takayama, K., Suzuki, T., Tsutsumi, S., Fujimura, T., Urano, T., Takahashi, S., Homma, Y., Aburatani, H., and Inoue, S. (2015). RUNX1, an androgen- and EZH2-regulated gene, has differential roles in AR-dependent and -independent prostate cancer. Oncotarget *6*, 2263-2276. 10.18632/oncotarget.2949.

Vahrenkamp, J.M., Yang, C.H., Rodriguez, A.C., Almomen, A., Berrett, K.C., Trujillo, A.N., Guillen, K.P., Welm, B.E., Jarboe, E.A., Janat-Amsbury, M.M., and Gertz, J. (2018). Clinical and Genomic Crosstalk between Glucocorticoid Receptor and Estrogen Receptor α In Endometrial Cancer. Cell Rep *22*, 2995-3005. 10.1016/j.celrep.2018.02.076.

Yamamoto, S., Takayama, K.i., Obinata, D., Fujiwara, K., Ashikari, D., Takahashi, S., and Inoue, S. (2019). Identification of new octamer transcription factor 1-target genes upregulated in castration-resistant prostate cancer. Cancer Science *110*, 3476-3485.

Zhang, G., Zhao, Y., Liu, Y., Kao, L.-P., Wang, X., Skerry, B., and Li, Z. (2016a). FOXA1 defines cancer cell specificity. Science Advances *2*, e1501473. doi:10.1126/sciadv.1501473.

Zhang, G., Zhao, Y., Liu, Y., Kao, L.P., Wang, X., Skerry, B., and Li, Z. (2016b). FOXA1 defines cancer cell specificity. Sci Adv *2*, e1501473. 10.1126/sciadv.1501473.
