# Supplementary material for: Cis-regulatory control of transcriptional timing and noise in response to estrogen
Source: bioRxiv. 2023 Mar 15:2023.03.14.532457. Preprint. [Version 1] doi: 10.1101/2023.03.14.532457 (PMC10054948; doi:10.1101/2023.03.14.532457)
Supplement: Supplementary file 1 [file media-1.pdf]

Supplementary Information

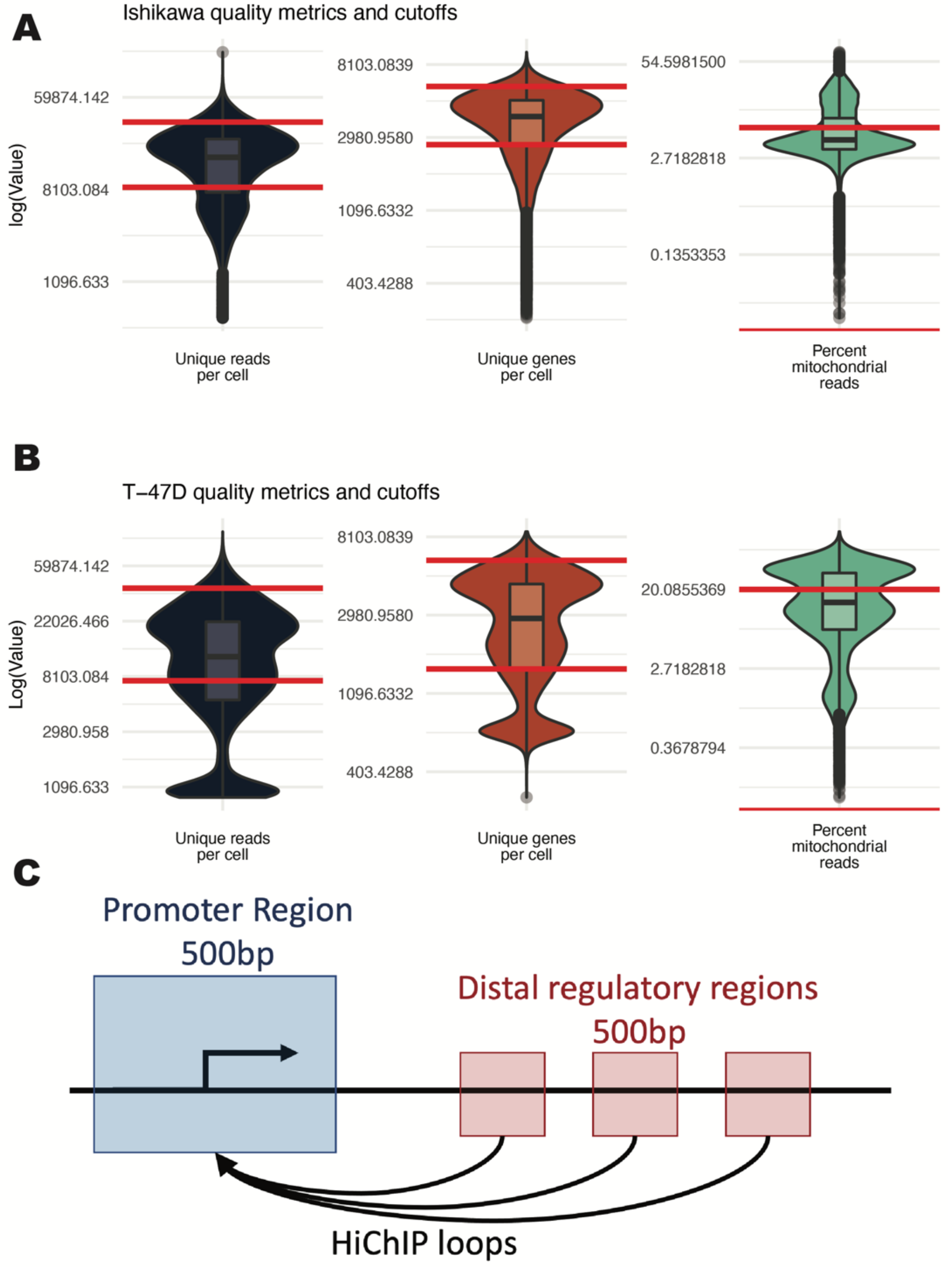

Supplementary Figure 1. QC plots for scRNA-seq and diagram of cis-regulatory model. (A-B) Distribution of reads, genes, and percent mitochondrial reads per cell that were used to filter cells for scRNA-seq analysis in Ishikawa (A) and T-47D (B). Cutoffs are shown with red lines. (C) Model shows how signal intensity was collected for promoters and enhancers using HiChIP loops to identify enhancers.

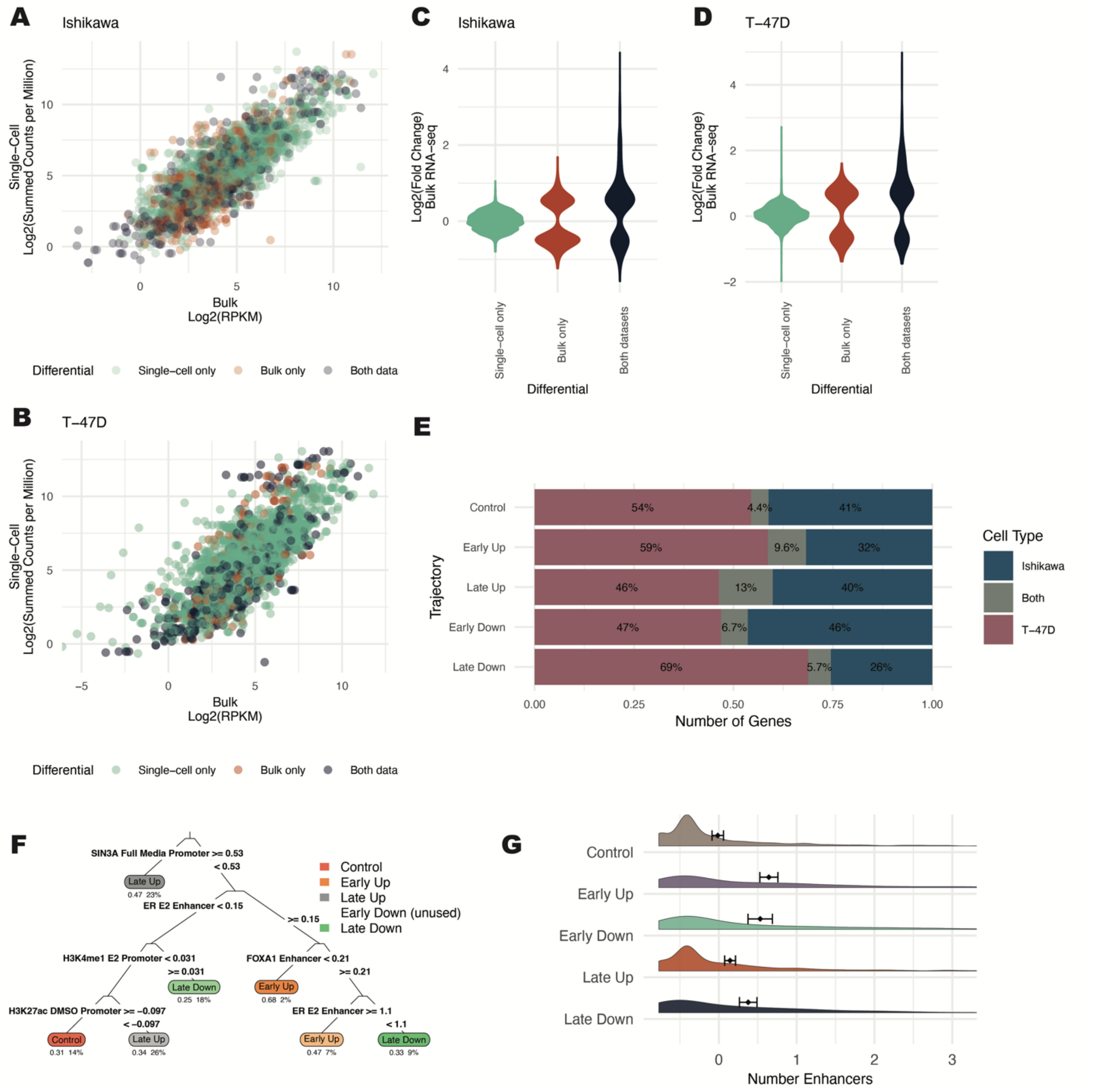

Supplementary Figure 2. Differential gene expression and determinants of trajectory. (A-B) Summed RPM from scRNA-seq are correlated to bulk RNA-seq RPM in both Ishikawa (A) and T-47D (B) cells, shown on a log scale. Colors show genes called as differential in either bulk RNA-seq, scRNA-seq, or both. (C-D) Log2 fold change distribution is shown for differential genes called by scRNA-seq analysis or bulk RNA-seq analysis in Ishikawa (C) and T-47D (D) cells. (E) Proportion of overlap between genes of each trajectory classification between cell types is shown. (F) Decision tree shows the hierarchy of classification for predicting gene expression trajectories. The top 4 layers are shown. (G) Distribution in number of enhancers (z-score) by trajectory is plotted.

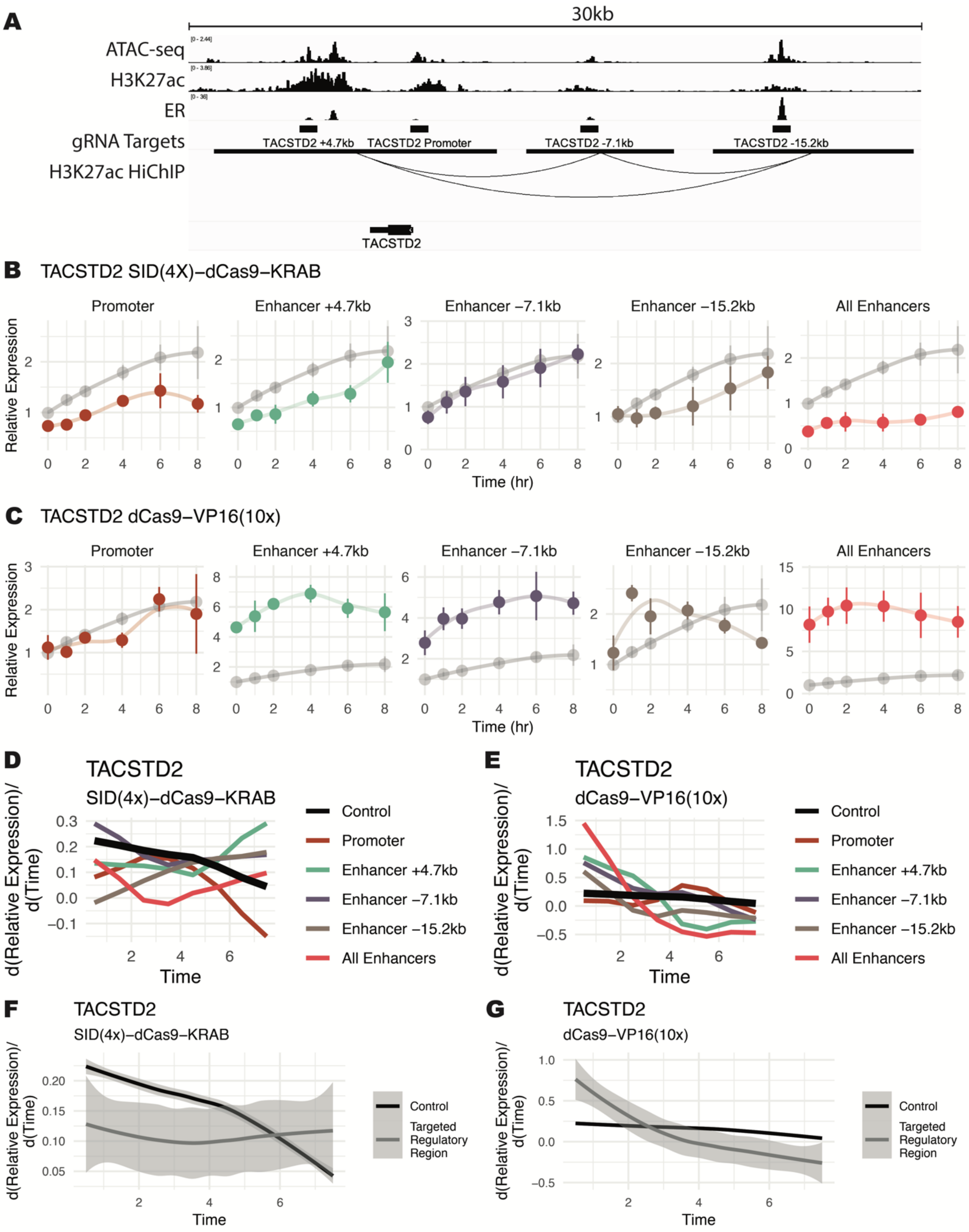
A
30kb
ATAC-seq
H3K27ac
ER
gRNA Targets
H3K27ac HiChIP
TACSTD2 +4.7kb
TACSTD2 Promoter
TACSTD2 -7.1kb
TACSTD2 -15.2kb
TACSTD2
B
TACSTD2 SID(4X)–dCas9–KRAB
Promoter
Enhancer +4.7kb
Enhancer −7.1kb
Enhancer −15.2kb
All Enhancers
Relative Expression
Time (hr)
C
TACSTD2 dCas9–VP16(10x)
Promoter
Enhancer +4.7kb
Enhancer −7.1kb
Enhancer −15.2kb
All Enhancers
Relative Expression
Time (hr)
D
TACSTD2
SID(4x)–dCas9–KRAB
d(Relative Expression)/d(Time)
Time
Control
Promoter
Enhancer +4.7kb
Enhancer −7.1kb
Enhancer −15.2kb
All Enhancers
E
TACSTD2
dCas9–VP16(10x)
d(Relative Expression)/d(Time)
Time
Control
Promoter
Enhancer +4.7kb
Enhancer −7.1kb
Enhancer −15.2kb
All Enhancers
F
TACSTD2
SID(4x)–dCas9–KRAB
d(Relative Expression)/d(Time)
Time
Control
Targeted Regulatory Region
G
TACSTD2
dCas9–VP16(10x)
d(Relative Expression)/d(Time)
Time
Control
Targeted Regulatory Region

Supplementary Figure 3. Synthetic transcription factors targeted to regulatory regions alter trajectories of *TGFA* and *PEG10*. (A) Expression trajectory of *TGFA* following E2 induction in Ishikawa cells is shown. Regulatory regions are targeted with SID(4x)-dCas9-KRAB. Non-targeting control is shown in gray. (B) *PEG10* gene expression trajectories in Ishikawa cells following estrogen inductions are modulated by dCas9-VP16(10x) targeted to regulatory regions. (C-D) Differential of loess regressions from panels A and B show expression rates of change when CREs near *TGFA* are targeted by SID(4x)-dCas9-KRAB (C) or *PEG10* CREs are targeted by dCas9-VP16(10x) (D). (E-F) Aggregate rates of change are shown for all regulatory regions targeted (grey) compared to control (black) for *TGFA* (E) and *PEG10* (F). The shaded region represents 95% confidence interval. (G) Validation of stable dCas9-VP16(10x) expressing Ishikawa cell line shows a control gene, *IL1RN,* being activated to previously observed levels (Ginley-Hidinger et al., 2019).

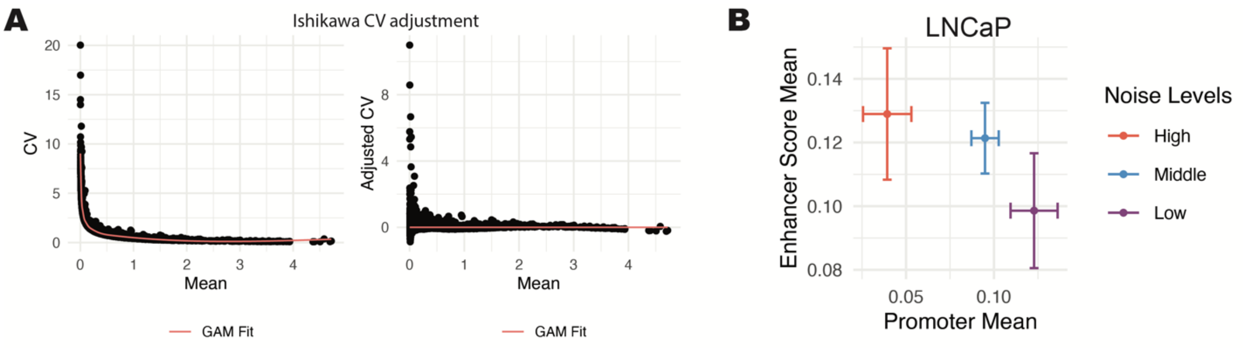

Supplementary Figure 4. Strong enhancers and weak promoters are associated with high levels of noise. (A) Calculated coefficient of variation (CV) is shown before (left panel) and after (right panel) adjustment using a generalized additive model regression fit. (B) Enhancer score averages vs promoter score averages are shown for noise levels in LNCaP cells. Averages are based on confirmed variables from a Boruta analysis. For a list of genomic data used see Table S3.

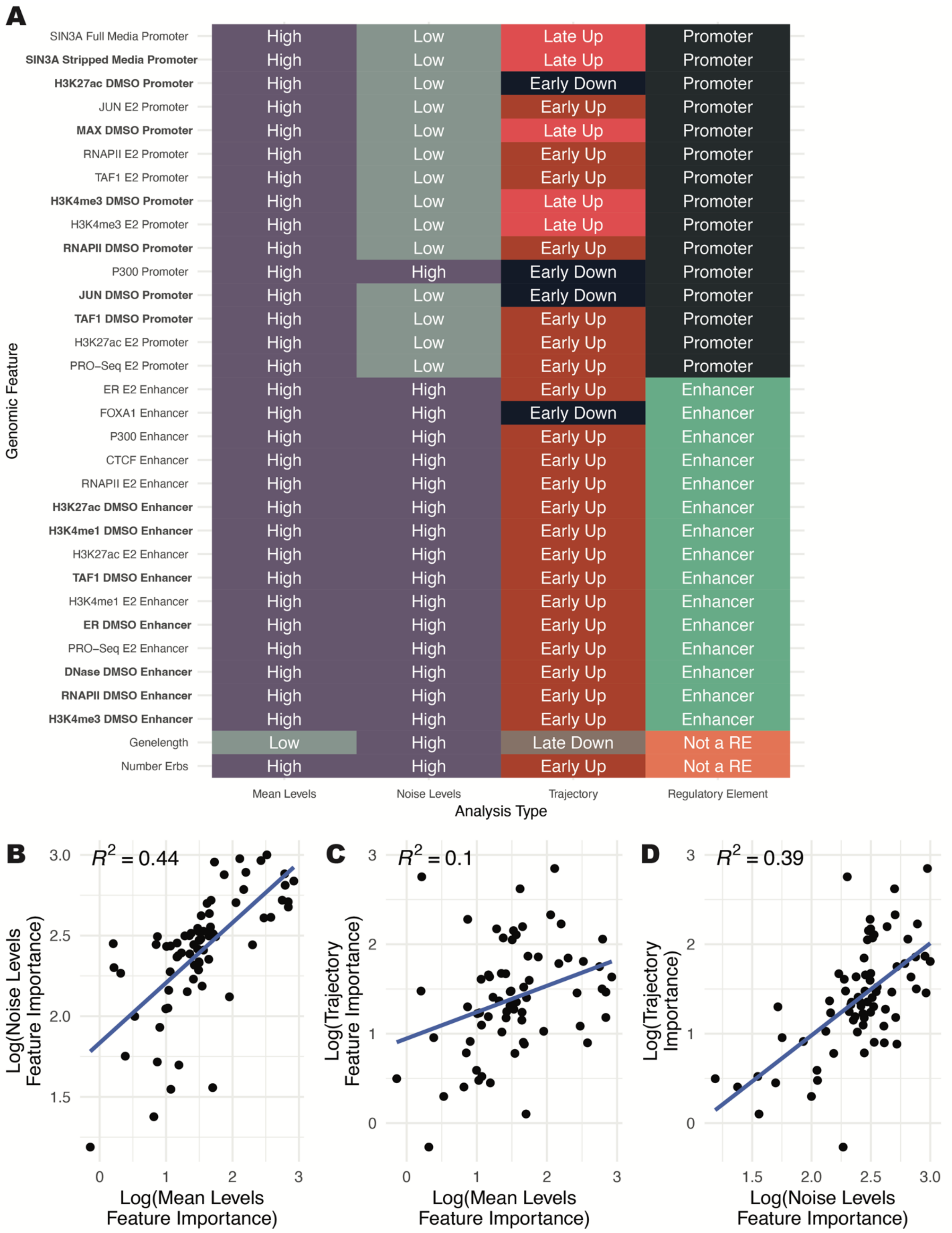
A
SIN3A Full Media Promoter | High | Low | Late Up | Promoter
SIN3A Stripped Media Promoter | High | Low | Late Up | Promoter
H3K27ac DMSO Promoter | High | Low | Early Down | Promoter
JUN E2 Promoter | High | Low | Early Up | Promoter
MAX DMSO Promoter | High | Low | Late Up | Promoter
RNAPII E2 Promoter | High | Low | Early Up | Promoter
TAF1 E2 Promoter | High | Low | Early Up | Promoter
H3K4me3 DMSO Promoter | High | Low | Late Up | Promoter
H3K4me3 E2 Promoter | High | Low | Late Up | Promoter
RNAPII DMSO Promoter | High | Low | Early Up | Promoter
P300 Promoter | High | High | Early Down | Promoter
JUN DMSO Promoter | High | Low | Early Down | Promoter
TAF1 DMSO Promoter | High | Low | Early Up | Promoter
H3K27ac E2 Promoter | High | Low | Early Up | Promoter
PRO–Seq E2 Promoter | High | Low | Early Up | Promoter
ER E2 Enhancer | High | High | Early Up | Enhancer
FOXA1 Enhancer | High | High | Early Down | Enhancer
P300 Enhancer | High | High | Early Up | Enhancer
CTCF Enhancer | High | High | Early Up | Enhancer
RNAPII E2 Enhancer | High | High | Early Up | Enhancer
H3K27ac DMSO Enhancer | High | High | Early Up | Enhancer
H3K4me1 DMSO Enhancer | High | High | Early Up | Enhancer
H3K27ac E2 Enhancer | High | High | Early Up | Enhancer
TAF1 DMSO Enhancer | High | High | Early Up | Enhancer
H3K4me1 E2 Enhancer | High | High | Early Up | Enhancer
ER DMSO Enhancer | High | High | Early Up | Enhancer
PRO–Seq E2 Enhancer | High | High | Early Up | Enhancer
DNase DMSO Enhancer | High | High | Early Up | Enhancer
RNAPII DMSO Enhancer | High | High | Early Up | Enhancer
H3K4me3 DMSO Enhancer | High | High | Early Up | Enhancer
Genelength | Low | High | Late Down | Not a RE
Number Erbs | High | High | Early Up | Not a RE
Genomic Feature
Mean Levels
Noise Levels
Trajectory
Regulatory Element
Analysis Type
B
$R^2 = 0.44$
Log(Noise Levels Feature Importance)
Log(Mean Levels Feature Importance)
C
$R^2 = 0.1$
Log(Trajectory Feature Importance)
Log(Mean Levels Feature Importance)
D
$R^2 = 0.39$
Log(Trajectory Importance)
Log(Noise Levels Feature Importance)

Supplementary Figure 5. Promoters and enhancers underlie different relationships between mean, noise, and trajectory. (A) Top 15 most important features for each regulatory element type are shown (promoter or enhancer), determined by average rank across all three analysis types. For each analysis, the classification group with the highest signal is displayed. Datasets shown in bold were performed in the absence of ER activation. (B-D) Pairwise correlation plots show the relationship between importance scores for the three analysis types. Each point represents a genomic feature. R^2 value is calculated using spearman correlation.

Table S1. gRNA sequences

gRNA Name	Sequence	PAM
PEG10 Promoter gRNA 1	GCCTTATATAGGTTGCACCG	AGG
PEG10 Promoter gRNA 2	CGCGTGGTGAGTATGCGGTG	AGG
PEG10 Promoter gRNA 3	GGAGTACGGGATTACCGAGG	GGG
PEG10 Promoter gRNA 4	TTAGTGGCCTGCGCGCGACG	TGG
PEG10 Enhancer +105kb gRNA 1	CATCTCCAGTTCAAACAGTG	AGG
PEG10 Enhancer +105kb gRNA 2	AAGCCCAGGTATATTAATGG	AGG
PEG10 Enhancer +105kb gRNA 3	TGACATAAACTAGCTGAGCA	GGG
PEG10 Enhancer +105kb gRNA 4	ACTTGAGCTTGGATCCAGCT	GGG
PEG10 Enhancer +158kb gRNA 1	GAGAGTTCCACTCTCGTGAA	TGG
PEG10 Enhancer +158kb gRNA 2	GACAGAGCAAGACTCCGTAA	AGG
PEG10 Enhancer +158kb gRNA 3	ATCTGCTGGAACCTTGATCA	TGG
PEG10 Enhancer +158kb gRNA 4	GAGGAGAGCCTCTTGAACCT	GGG
PEG10 Enhancer +45kb gRNA 1	GGAATATGCTCATAACCACA	AGG
PEG10 Enhancer +45kb gRNA 2	CTCTTAATCTCCCTTCACCA	AGG
PEG10 Enhancer +45kb gRNA 3	TCCTATTTCCTGTTGCCCTG	GGG
PEG10 Enhancer +45kb gRNA 4	CCTAATTCCAGGCTCACCAC	AGG
PEG10 Enhancer +-305kb gRNA 1	GGCATACTGGAATAAACATG	AGG
PEG10 Enhancer +-305kb gRNA 2	TGAGAGACTCACTTGTCACT	AGG
PEG10 Enhancer +-305kb gRNA 3	ACACTTCTAGAAGGCCCACT	AGG
PEG10 Enhancer +-305kb gRNA 4	TAAAGAAGGTATAAGATCCA	TGG
TACSTD2 Promoter gRNA 1	GGTAGAGTATAAGAGCCGGA	GGG
TACSTD2 Promoter gRNA 2	TGTTCTGATCCTATCGCGGG	CGG
TACSTD2 Promoter gRNA 3	GAGAACCGATACTCACCTGT	AGG
TACSTD2 Promoter gRNA 4	TAACACCAGTCCTGACAGGT	AGG
TACSTD2 Enhancer +4.7kb gRNA 1	CATCAGGAGACTAACCAGGG	AGG
TACSTD2 Enhancer +4.7kb gRNA 2	GAAGACACTGAAGCTCATAG	AGG
TACSTD2 Enhancer +4.7kb gRNA 3	AAGCCCTGGTGACCTACACA	AGG
TACSTD2 Enhancer +4.7kb gRNA 4	CAGTGAACCAGTGAACTGTA	AGG
TACSTD2 Enhancer -7.1kb gRNA 1	TGTAAGGGCTTTACACTCTG	GGG
TACSTD2 Enhancer -7.1kb gRNA 2	AGCAGAACTGTACACATCAC	AGG
TACSTD2 Enhancer -7.1kb gRNA 3	GCACCGTGACTCATCAGTGT	GGG
TACSTD2 Enhancer -7.1kb gRNA 4	GATGTGTGATGCCCTGTTAA	GGG
TACSTD2 Enhancer -15.2kb gRNA 1	TGGATTCAAGATCGAAACCC	TGG
TACSTD2 Enhancer -15.2kb gRNA 2	TGACTTCATCAGATGGAAAG	TGG
TACSTD2 Enhancer -15.2kb gRNA 3	GACATGAACGAGTCCTTGGA	AGG
TACSTD2 Enhancer -15.2kb gRNA 4	TGGGCAGGCTGATTAATGAG	GGG
TGFA Enhancer +44kb gRNA 1	TGAGTACCCAGCTCATGCA	AGG

TGFA Enhancer +44kb gRNA 2	GGGCATTACTCAGGCGCCC	GGG
TGFA Enhancer +44kb gRNA 3	CCCTGGTTAAGAAGTTGTT	GGG
TGFA Enhancer +44kb gRNA 4	GACAGGTGTTCTAAGCTAT	AGG
TGFA Promoter gRNA 1	CCCCGGTCGCCGAGTGGCG	AGG
TGFA Promoter gRNA 2	GAGCGCACCGAGCAGGGCG	CGG
TGFA Promoter gRNA 3	GCACGGCGCTGGCAGTCGG	GGG
TGFA Promoter gRNA 4	TAGGTAACGGGTGCCCGGG	CGG
TGFA Enhancer -43kb gRNA 1	GGAAGCGGGATGCAGACCT	GGG
TGFA Enhancer -43kb gRNA 2	CAAGACATCAGGCTTCCTG	GGG
TGFA Enhancer -43kb gRNA 3	GTTCACCACAGCGTGTCAA	GGG
TGFA Enhancer -43kb gRNA 4	CCAGGATCTGCCACCCCTG	TGG
TGFA Enhancer -53kb gRNA 1	CACCGAATCAACTGAGGTC	AGG
TGFA Enhancer -53kb gRNA 2	GGAGTTCCTACAATCACAG	AGG
TGFA Enhancer -53kb gRNA 3	AAGACAACGGATGTGTCCG	TGG
TGFA Enhancer -53kb gRNA 4	AGAAGCCCGAACCCAGAGA	AGG
TGFA Enhancer -54kb gRNA 1	GAACGGGGACTTGAGGTAT	TGG
TGFA Enhancer -54kb gRNA 2	AGCATCACGCTGAGAATGG	GGG
TGFA Enhancer -54kb gRNA 3	GGACATCCACTTGAAAAGC	TGG
TGFA Enhancer -54kb gRNA 4	TAAAATGAGTGCTCTAGGC	AGG
TGFA Enhancer -62kb gRNA 1	AGGGCTACCTAACTGCAAT	AGG
TGFA Enhancer -62kb gRNA 2	ATTGTCTGAACTCCGGATG	TGG
TGFA Enhancer -62kb gRNA 3	TCAGGGAGTGTTGCAACCT	TGG
TGFA Enhancer -62kb gRNA 4	GTATCACAGCGAAGCGGGT	GGG

Table S2. qPCR primers

TACSTD2_F	ACAACGATGGCCTCTACGAC
TACSTD2_R	GTCCAGGTCTGAGTGGTTGAA
PEG10_F	GAGCACCAGGGATTTCTCAGT
PEG10_R	GGTAGTTGTGCATCAGGTAGTG
TGFA_F	AGGTCCGAAAACACTGTGAGT
TGFA_R	AGCAAGCGGTTCTTCCCTTC
IL1RN_F	GGAATCCATGGAGGGAAGAT
IL1RN_R	TGTTCTCGCTCAGGTCAGTG
dCas9_F	GTGACCGAGGGAATGAGAAA
dCas9_R	AGCTGCTTCACGGTCACTTT

Table S3. Genomic Source Information

* - indicates experiments conducted for this study. See Data Availability.

Cell Line	Genomic Feature	Reference	Accession
Ishikawa	CTCF	(ENCODE, 2012)	GSE32465
Ishikawa	Dnase	(ENCODE, 2012)	GSE32970
Ishikawa	ER	(ENCODE, 2012; Rodriguez et al., 2020)	GSE32465; GSE129803
Ishikawa	FOXA1	(ENCODE, 2012)	GSE32465
Ishikawa	GC Content	(Kent et al., 2002)	
Ishikawa	Gene length	(Kent *et al.*, 2002)	
Ishikawa	H3K27ac	(Vahrenkamp et al., 2018)	GSE109893
Ishikawa	H3K27me3	*	
Ishikawa	H3K36me3	*	
Ishikawa	H3K4me1	*	
Ishikawa	H3K4me3	*	
Ishikawa	H3K9me3	(Carleton et al., 2017)	GSE99906
Ishikawa	JUN	*	
Ishikawa	LSD1	*	
Ishikawa	MAX	*	
Ishikawa	MYC	*	
Ishikawa	Number Enhancers	*	
Ishikawa	Number ERBS	*	
Ishikawa	p300	(ENCODE, 2012)	GSE32465
Ishikawa	PRO-seq	*	
Ishikawa	RARA	*	
Ishikawa	RNAPII	(Carleton *et al.*, 2017; ENCODE, 2012)	GSE32465; GSE99906
Ishikawa	SIN3A	*	

Ishikawa	TAF1	*	
Ishikawa	scRNA-seq	*	
Ishikawa	HiChIP	*	
T-47D	CTCF	(ENCODE, 2012)	GSE32465
T-47D	Dnase	(ENCODE, 2012)	GSE32970
T-47D	ER	(ENCODE, 2012)	GSE32465
T-47D	FOXA1	(ENCODE, 2012)	GSE32465
T-47D	GC Content	(Kent *et al.*, 2002)	
T-47D	Gene length	(Kent *et al.*, 2002)	
T-47D	H3K27ac	(Carleton et al., 2020; Zhang et al., 2016b)	GSE63109; GSE147141
T-47D	H3K27me3	*	
T-47D	H3K36me3	(Zhang et al., 2016a)	GSE63109
T-47D	H3K4me1	*	
T-47D	H3K4me3	*	
T-47D	H3K9me3	(Zhang *et al.*, 2016a)	GSE63109
T-47D	JUN	*	
T-47D	LSD1	*	
T-47D	MAX	*	
T-47D	MYC	*	
T-47D	Number Enhancers	*	
T-47D	Number ERBS	*	
T-47D	p300	(ENCODE, 2012)	GSE32465
T-47D	PRO-seq	*	
T-47D	RARA	*	
T-47D	RNAPII	*	
T-47D	SIN3A	*	
T-47D	TAF1	*	
T-47D	scRNA-seq	*	
T-47D	HiChIP	*	
LNCaP	H3ac	(Takayama et al., 2015; Yamamoto et al., 2019)	GSE62492
LNCaP	AR	(Takayama *et al.*, 2015; Yamamoto *et al.*, 2019)	GSE62492
LNCaP	CHD1	(Metzger et al., 2016)	GSE64530
LNCaP	ETV1	(Chen et al., 2013)	GSE47120
LNCaP	FOXP1	(Takayama *et al.*, 2015; Yamamoto *et al.*, 2019)	GSE62492
LNCaP	H3K27ac	(Taberlay et al., 2016)	GSE73785

LNCaP	H3K27me3	(Takayama *et al.*, 2015; Yamamoto *et al.*, 2019)	GSE62492
LNCaP	H3K4me1	(Takayama *et al.*, 2015; Yamamoto *et al.*, 2019)	GSE62492
LNCaP	H3K4me3	(Takayama *et al.*, 2015; Yamamoto *et al.*, 2019)	GSE62492
LNCaP	LSD1	(Metzger *et al.*, 2016)	GSE64530
LNCaP	RUNX1	(Takayama *et al.*, 2015; Yamamoto *et al.*, 2019)	GSE62492
LNCaP	scRNA-seq	(Taavitsainen et al., 2021)	GSE168668
LNCaP	HiChIP	*	

Supplemental References

Carleton, J.B., Berrett, K.C., and Gertz, J. (2017). Multiplex Enhancer Interference Reveals Collaborative Control of Gene Regulation by Estrogen Receptor α-Bound Enhancers. Cell Syst *5*, 333-344.e335. 10.1016/j.cels.2017.08.011.

Carleton, J.B., Ginley-Hidinger, M., Berrett, K.C., Layer, R.M., Quinlan, A.R., and Gertz, J. (2020). Regulatory sharing between estrogen receptor α bound enhancers. Nucleic Acids Research *48*, 6597-6610. 10.1093/nar/gkaa454.

Chen, Y., Chi, P., Rockowitz, S., Iaquinta, P.J., Shamu, T., Shukla, S., Gao, D., Sirota, I., Carver, B.S., and Wongvipat, J. (2013). ETS factors reprogram the androgen receptor cistrome and prime prostate tumorigenesis in response to PTEN loss. Nature medicine *19*, 1023-1029.

ENCODE, C. (2012). An integrated encyclopedia of DNA elements in the human genome. Nature *489*, 57-74. 10.1038/nature11247.

Ginley-Hidinger, M., Carleton, J.B., Rodriguez, A.C., Berrett, K.C., and Gertz, J. (2019). Sufficiency analysis of estrogen responsive enhancers using synthetic activators. Life Science Alliance *2*, e201900497. 10.26508/lsa.201900497.

Kent, W.J., Sugnet, C.W., Furey, T.S., Roskin, K.M., Pringle, T.H., Zahler, A.M., and Haussler, D. (2002). The human genome browser at UCSC. Genome research *12*, 996-1006.

Metzger, E., Willmann, D., McMillan, J., Forne, I., Metzger, P., Gerhardt, S., Petroll, K., Von Maessenhausen, A., Urban, S., and Schott, A.-K. (2016). Assembly of methylated KDM1A and CHD1 drives androgen receptor–dependent transcription and translocation. Nature structural & molecular biology *23*, 132-139.

Rodriguez, A.C., Vahrenkamp, J.M., Berrett, K.C., Clark, K.A., Guillen, K.P., Scherer, S.D., Yang, C.-H., Welm, B.E., Janát-Amsbury, M.M., and Graves, B.J. (2020). ETV4 is necessary for estrogen signaling and growth in endometrial cancer cells. Cancer research *80*, 1234-1245.

Taavitsainen, S., Engedal, N., Cao, S., Handle, F., Erickson, A., Prekovic, S., Wetterskog, D., Tolonen, T., Vuorinen, E.M., Kiviaho, A., et al. (2021). Single-cell ATAC and RNA sequencing reveal pre-existing and persistent cells associated with prostate cancer relapse. Nat Commun *12*, 5307. 10.1038/s41467-021-25624-1.

Taberlay, P.C., Achinger-Kawecka, J., Lun, A.T., Buske, F.A., Sabir, K., Gould, C.M., Zotenko, E., Bert, S.A., Giles, K.A., and Bauer, D.C. (2016). Three-dimensional disorganization of the cancer genome occurs coincident with long-range genetic and epigenetic alterations. Genome research *26*, 719-731.

Takayama, K., Suzuki, T., Tsutsumi, S., Fujimura, T., Urano, T., Takahashi, S., Homma, Y., Aburatani, H., and Inoue, S. (2015). RUNX1, an androgen- and EZH2-regulated gene, has differential roles in AR-dependent and -independent prostate cancer. Oncotarget *6*, 2263-2276. 10.18632/oncotarget.2949.

Vahrenkamp, J.M., Yang, C.H., Rodriguez, A.C., Almomen, A., Berrett, K.C., Trujillo, A.N., Guillen, K.P., Welm, B.E., Jarboe, E.A., Janat-Amsbury, M.M., and Gertz, J. (2018). Clinical and Genomic Crosstalk between Glucocorticoid Receptor and Estrogen Receptor α In Endometrial Cancer. Cell Rep *22*, 2995-3005. 10.1016/j.celrep.2018.02.076.

Yamamoto, S., Takayama, K.i., Obinata, D., Fujiwara, K., Ashikari, D., Takahashi, S., and Inoue, S. (2019). Identification of new octamer transcription factor 1-target genes upregulated in castration-resistant prostate cancer. Cancer Science *110*, 3476-3485.

Zhang, G., Zhao, Y., Liu, Y., Kao, L.-P., Wang, X., Skerry, B., and Li, Z. (2016a). FOXA1 defines cancer cell specificity. Science Advances *2*, e1501473. doi:10.1126/sciadv.1501473.

Zhang, G., Zhao, Y., Liu, Y., Kao, L.P., Wang, X., Skerry, B., and Li, Z. (2016b). FOXA1 defines cancer cell specificity. Sci Adv *2*, e1501473. 10.1126/sciadv.1501473.
